# Global Mapping of Cell Type–Specific Open Chromatin by FAIRE-seq Reveals the Regulatory Role of the NFI Family in Adipocyte Differentiation

**DOI:** 10.1371/journal.pgen.1002311

**Published:** 2011-10-20

**Authors:** Hironori Waki, Masahiro Nakamura, Toshimasa Yamauchi, Ken-ichi Wakabayashi, Jing Yu, Lisa Hirose-Yotsuya, Kazumi Take, Wei Sun, Masato Iwabu, Miki Okada-Iwabu, Takanori Fujita, Tomohisa Aoyama, Shuichi Tsutsumi, Kohjiro Ueki, Tatsuhiko Kodama, Juro Sakai, Hiroyuki Aburatani, Takashi Kadowaki

**Affiliations:** 1 Department of Diabetes and Metabolic Diseases, Graduate School of Medicine, University of Tokyo, Tokyo, Japan; 2 Functional Regulation of Adipocytes, Graduate School of Medicine, University of Tokyo, Tokyo, Japan; 3 Genome Science Division, Laboratory of Systems Biology and Medicine, Research Center for Advanced Science and Technology, University of Tokyo, Tokyo, Japan; 4 Department of Integrated Molecular Science on Metabolic Diseases, 22nd Century Medical and Research Center, University of Tokyo, Tokyo, Japan; 5 Molecular Medicinal Sciences on Metabolic Regulation, Graduate School of Medicine, University of Tokyo, Tokyo, Japan; 6 Systems Biology and Medicine Division, Laboratory of Systems Biology and Medicine, Research Center for Advanced Science and Technology, University of Tokyo, Tokyo, Japan; 7 Metabolic Medicine Division, Laboratory of Systems Biology and Medicine, Research Center for Advanced Science and Technology, University of Tokyo, Tokyo, Japan; The University of North Carolina at Chapel Hill, United States of America

## Abstract

Identification of regulatory elements within the genome is crucial for understanding the mechanisms that govern cell type–specific gene expression. We generated genome-wide maps of open chromatin sites in 3T3-L1 adipocytes (on day 0 and day 8 of differentiation) and NIH-3T3 fibroblasts using formaldehyde-assisted isolation of regulatory elements coupled with high-throughput sequencing (FAIRE-seq). FAIRE peaks at the promoter were associated with active transcription and histone modifications of H3K4me3 and H3K27ac. Non-promoter FAIRE peaks were characterized by H3K4me1+/me3-, the signature of enhancers, and were largely located in distal regions. The non-promoter FAIRE peaks showed dynamic change during differentiation, while the promoter FAIRE peaks were relatively constant. Functionally, the adipocyte- and preadipocyte-specific non-promoter FAIRE peaks were, respectively, associated with genes up-regulated and down-regulated by differentiation. Genes highly up-regulated during differentiation were associated with multiple clustered adipocyte-specific FAIRE peaks. Among the adipocyte-specific FAIRE peaks, 45.3% and 11.7% overlapped binding sites for, respectively, PPARγ and C/EBPα, the master regulators of adipocyte differentiation. Computational motif analyses of the adipocyte-specific FAIRE peaks revealed enrichment of a binding motif for nuclear family I (NFI) transcription factors. Indeed, ChIP assay showed that NFI occupy the adipocyte-specific FAIRE peaks and/or the PPARγ binding sites near PPARγ, C/EBPα, and aP2 genes. Overexpression of NFIA in 3T3-L1 cells resulted in robust induction of these genes and lipid droplet formation without differentiation stimulus. Overexpression of dominant-negative NFIA or siRNA–mediated knockdown of NFIA or NFIB significantly suppressed both induction of genes and lipid accumulation during differentiation, suggesting a physiological function of these factors in the adipogenic program. Together, our study demonstrates the utility of FAIRE-seq in providing a global view of cell type–specific regulatory elements in the genome and in identifying transcriptional regulators of adipocyte differentiation.

## Author Summary

Humans consist of a few hundred types of specialized-function cells. Spatial and temporal transcriptional regulation of genes is essential for manifestation of cellular phenotypes. Identification of regulatory regions in the genome is central to understanding the mechanism of cell type–specific gene regulation. Recently developed high-throughput sequencing technology and computational analyses allow genome-wide investigation of the genome's chromatin structure. Using the FAIRE-seq technique, we identified the genome's open chromatin regions, which harbor regulatory elements in adipocytes. Open chromatin regions distal to genes' transcription start sites significantly differ among cell types. Multiple cell type–specific open chromatin regions exist near genes regulated during adipocyte differentiation. Computational motif analysis of adipocyte-specific open chromatin regions revealed enrichment of a binding motif for the NFI transcription factor family. These factors bind to the regulatory elements near adipogenic PPARγ, C/EBPα, and aP2 genes and regulate their expression. Overexpression of NFIA in 3T3-L1 cells resulted in robust induction of these genes and lipid droplet formation without differentiation stimulus and knockdown of NFIA or NFIB significantly suppressed both induction of genes and lipid accumulation during differentiation. Our study demonstrates the utility of FAIRE-seq in providing a global view of regulatory elements and in identifying transcriptional regulators of cellular functions.

## Introduction

Sequencing allowed identification and mapping of the human genome [1]. Transcriptional regulation of genes is essential for manifesting cellular phenotypes and complex biological processes. Coordinated actions of transcription factors and cofactors on regulatory DNA sequences produce transcriptional activation of the eukaryotic gene. Therefore, identification and mapping of the genome's regulatory elements is critical for understanding how cell-type-selective regulation of genes in the genome is achieved.

Traditionally, regulatory elements have been identified by DNase I hypersensitivity assay combined with Southern blot analysis [2]. That assay coupled with microarray or high-throughput sequencing (DNase-Chip or DNase-seq) were effectively applied in genome-wide identification of open chromatin regions [3], [4], [5], [6]. Lieb and his colleagues recently developed formaldehyde-assisted isolation of regulatory elements (FAIRE) as a simple procedure to isolate nucleosome-depleted DNA from chromatin [7], [8]. FAIRE detects open chromatin structure much the way the DNase I hypersensitivity assay does [8], [9]—but with advantages, like obviating the need for clean nuclei preparation and laborious enzyme titrations [7], [8]. Coupled with high-throughput sequencing (FAIRE-seq), FAIRE allows unbiased identification of potential regulatory elements without requiring prior knowledge of (or about) binding factors. FAIRE-seq's genome-wide detection of open chromatin genomic regions in human pancreatic islets was successfully used to determine a causal single nucleotide polymorphism in loci associated with type 2 diabetes development in genome-wide association studies [10].

The adipocyte is central in controlling energy balance and whole-body glucose and lipid homeostasis [11]. Advances in adipocyte research have shown that adipose tissue stores excess energy *and* secretes hormones and metabolites to communicate with other organs, maintaining systemic metabolic homeostasis [12]. Peroxisome proliferator-activated receptor gamma (PPARγ; NR1C3) is both necessary [13], [14], [15] and sufficient [16] for adipocyte differentiation. Necessary for both development and maintenance of mature adipocytes, PPARγ is crucial in systemic glucose and lipid homeostasis [13], [14], [15], [17], and, importantly, is the molecular target of thiazolidinediones, widely prescribed for obese diabetics [18]. C/EBPα-β-δ act with PPARγ, forming the adipogenic transcription cascade [19]. C/EBPβ and δ are induced by adipogenic stimulus, inducing PPARγ, which activates expression of C/EBPα, which binds and further activates expression of PPARγ, providing a positive regulatory loop [11], [20]. Genome-wide approaches now dissect the transcriptional mechanisms of adipocyte differentiation. ChIP-chip or ChIP-seq studies of adipogenic regulators [21], [22], [23], [24], [25], [26], [27], [28], [29] have provided valuable mechanistic insights into adipogenic transcription never before gained by conventional experiments: New concepts include co-localization of PPARγ and cell type–specific transcription factors [27], low conservation rate of PPARγ binding sites between murine and human adipocytes [28] and the role of C/EBPβ as a pioneer factor that establishes “hot spots” where multiple adipogenic regulators cooperatively work in the very early stage of differentiation [6].

Our study took an unbiased approach to mapping adipocyte-specific regulatory elements in the genome by using FAIRE in 3T3-L1 adipocytes (on day 0 and day 8 of differentiation) and NIH-3T3 fibroblasts. We show that the FAIRE peaks contain regulatory elements such as promoters, enhancers and insulators, and that adipocyte-specific non-promoter FAIRE peaks are functionally linked to genes regulated during differentiation—about half these peaks being overlapped by PPARγ. We show that highly regulated genes in adipocyte differentiation are associated with clusters of multiple adipocyte-specific non-promoter FAIRE peaks. Furthermore, because FAIRE does not require a prioi knowledge of bound transcription factors, we could employ computational motif analyses of DNA sequences from the adipocyte-specific FAIRE peaks in an unbiased manner and identify a motif for nuclear family I (NFI) transcription factors in addition to motifs for PPAR and C/EBPs. We show the functional role of NFIA and NFIB in adipocyte differentiation. We demonstrate the utility of FAIRE-seq both in providing a global view of cell type–specific *cis*-regulatory elements in the genome and identifying transcriptional regulators of adipocyte differentiation.

## Results

### Genome-Wide Profiling of Open Chromatin Regions in 3T3-L1 Adipocytes by FAIRE-seq

Regulatory elements in the genome are characterized by open chromatin structures accessible to regulatory factors [30]. To explore genome-wide changes in open chromatin conformation during adipocyte differentiation, we used FAIRE—a method of isolating genomic regions depleted of nucleosomes [7]—combined with high-throughput sequencing (FAIRE-seq) to identify open chromatin sites in the adipogenic cell line 3T3-L1 before (day 0) and after (day 8) differentiation and in NIH-3T3 fibroblasts, which cannot differentiate into adipocytes. This approach identified in the genome 37,781 FAIRE peaks in 3T3-L1 on day 0 and 26,611 on day 8, plus 36,111 in NIH-3T3 cells—all, with a false discovery rate of <10^−4^. By using ChIP-seq analyses, we also generated genome-wide maps of binding sites for PPARγ, the master regulator of adipocyte differentiation, for RXRα, its heterodimer partner, for histone H3 lysine 4 trimethylation (H3K4me3), and for CCCTC-binding factor (CTCF) [31].

**Figure 1:**
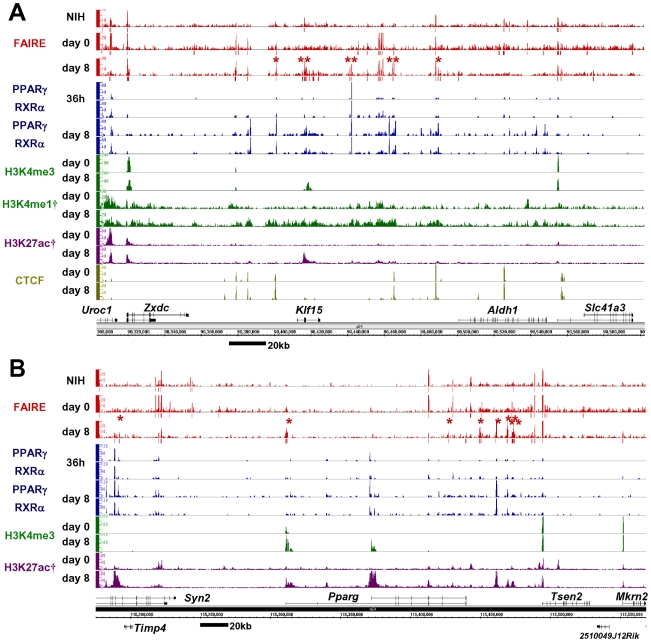
Genome-wide profiling of open chromatin regions by FAIRE-seq in 3T3-L1 adipocyte differentiation. Open chromatin regions detected by FAIRE-seq were observed in both promoter and non-promoter regions. The non-promoter FAIRE peaks were associated with the binding of PPARγ/RXRα or CTCF, and with the enhancer signature H3K4me1(+)/me3(−) and H3K27ac modification—while the promoter FAIRE peaks were associated with H3K4me3 and H3K27ac modification. Bars below the FAIRE peaks data represent statistically significant FAIRE positive peaks (FDR<10^−4^). Red asterisks indicate the adipocyte-specific FAIRE peaks on day 8 (see Figure 2B for definition). Multiple adipocyte-specific FAIRE peaks were located within genomic regions near *Klf15* (A) and *Pparg* (B) in 3T3-L1 adipocytes. Data marked (†) were obtained from Mikkelsen et al. [28] (GSE20752).

Figure 1 shows a representative map of results generated near *Klf15* and *Pparg*, both transcription factors up-regulated by differentiation, and both important in adipocyte differentiation [16], [32]. Consistent with previous observations [10], 28% of the FAIRE peaks were detected near the transcription start sites (TSSs ±500 bp) of RefSeq genes [33] and are referred to as promoter FAIRE peaks (Figure S1A), while 72% were located outside known TSSs, and are referred to as non-promoter FAIRE peaks. Notably, only 8% of the non-promoter FAIRE peaks were located in a −5 kb proximal promoter region while the majority of non-promoter FAIRE peaks were located in introns and distal regions (Figure S1A). Average profiling revealed that a FAIRE signal, H3K4me3 and histone H3 lysine 27 acetylation (H3K27ac) were observed at TSSs of actively transcribed genes (Figure S1B and S1D). On the other hand, non-promoter FAIRE peaks were accompanied by monomodal enrichment of H3K4me1 and were devoid of H3K4me3 enrichment, a condition described as the signature of enhancers [34], [35] (Figure S1D). CTCF binding sites are important in insulator function and high-order chromatin structure [31]. The CTCF binding sites in our study (day 0 or day 8) were largely overlapped by those in a study by Mikkelsen (day 0 or day 7) [28] (86.3% and 88.5%, respectively). CTCF binding accounted for about one fifth of either the promoter or non-promoter FAIRE peaks (Figure 1 and Figure S1C). Collectively, these data suggest that the open chromatin sites identified by FAIRE-seq show characteristics of regulatory elements such as promoter, enhancer and insulator.

### Analysis of Differentiation-Dependent Non-Promoter FAIRE Peaks

**Figure 2:**
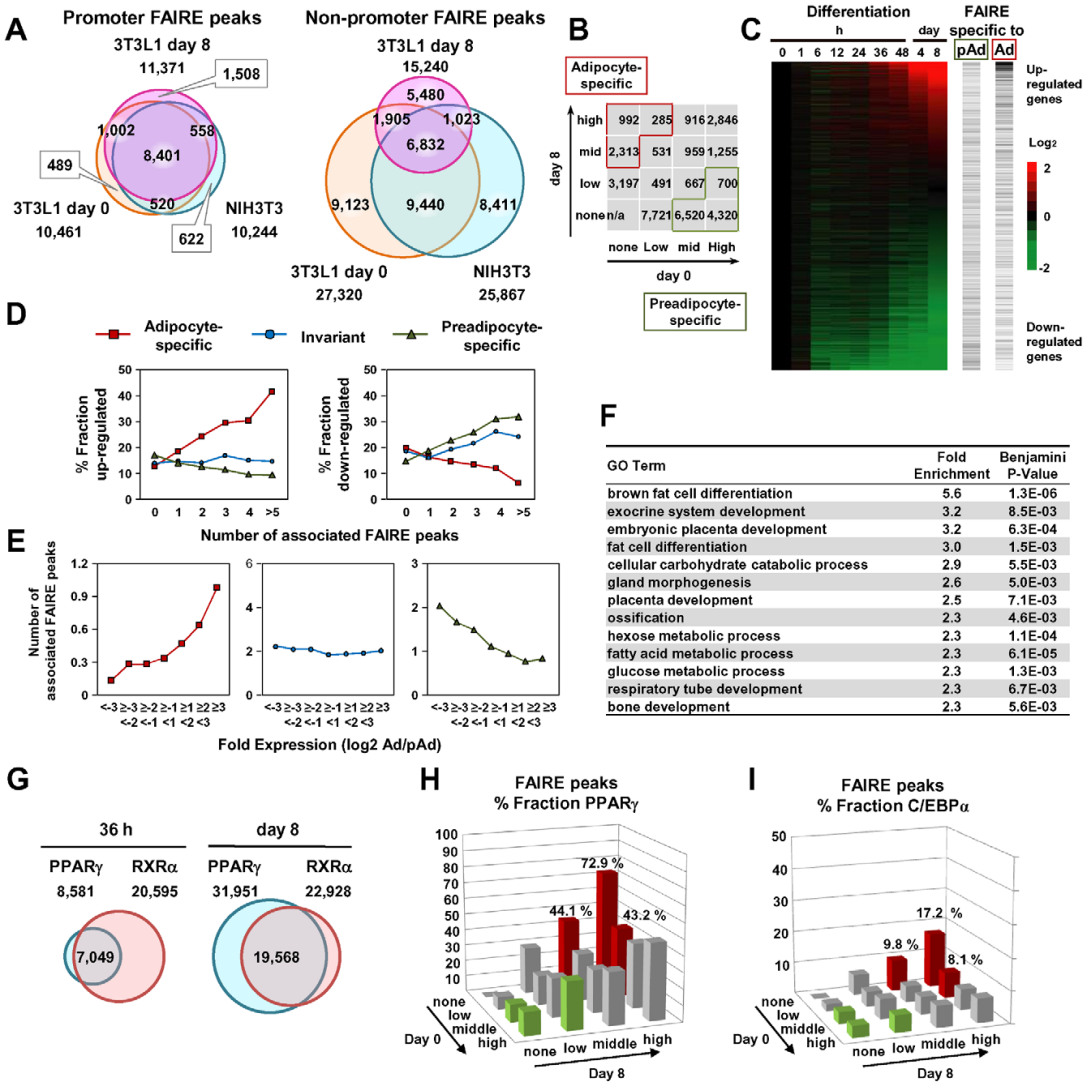
Cell type– and differentiation-dependent FAIRE peaks. (A) Venn diagrams comparing the FAIRE peaks among 3T3-L1 (day 0), 3T3-L1 (day 8) and NIH-3T3 at promoter (+/−500 bp from RefSeq TSS) and non-promoter regions. The promoter FAIRE peaks were relatively constant among the three cell groups while the non-promoter FAIRE peaks were highly variable. (B) The FAIRE peaks in 3T3-L1 (day 0 or day 8) were divided into tertiles by peak height and adipocyte- (red boxes) and preadipocyte-specific (green boxes) FAIRE peaks, and were defined as indicated. (C) A heat map showing enrichment of the adipocyte- and preadipocyte-specific FAIRE peaks in the vicinity (+/−25 kb from TSS) of genes up-regulated or down-regulated during differentiation. The horizontal bars in the two right panels indicate each gene with Ad or pAd FAIRE peaks in the vicinity (+/−25 kb from TSS). (D) Fractions of genes that were up-regulated (left) or down-regulated (right) more than two-fold during differentiation among genes that had the indicated number of adipocyte- (red), preadipocyte-specific (green) or invariant (blue) FAIRE peaks. (E) The number of the adipocyte- (red), preadipocyte-specific (green) or invariant (blue) FAIRE peaks associated with genes that were stratified by the ratio of the expression levels between preadipocytes and adipocytes. Each FAIRE peak was defined as associated with the nearest gene in analyses (D) and (E). (F) Ontology analysis by DAVID of genes associated (+/−25 kb from TSS) with adipocyte-specific FAIRE peaks [13]. (G) Venn diagrams showing the numbers and overlap of the binding sites for PPARγ and RXRα in 3T3-L1, day 0 and day 8. (H, I) Fractions of the non-promoter FAIRE peaks that overlap PPARγ binding sites (day 8) (H) or C/EBPα binding sites (Schmidt et al., GSE27450 [86]) (I). PPARγ and C/EBPα represented 45.3% and 11.7% of the adipocyte-specific FAIRE peaks (average of red bars).

We next compared the FAIRE peaks in 3T3-L1 cells on day 0 and day 8 and in NIH-3T3 cells. The promoter FAIRE peaks were relatively constant among the three groups. Over 70% of those peaks on day 0 and day 8 3T3-L1 cells and in NIH-3T3 cells were shared by all three groups (Figure 2A). In contrast, non-promoter FAIRE peaks showed dynamic change. The three groups shared only 25%, 45%, and 26% of non-promoter FAIRE peaks in, respectively, day 0 and day 8 3T3-L1 cells and NIH-3T3 cells. This contrasts with an invariable biding pattern of CTCF in the non-promoter regions; in 3T3-L1 cells, 89.5% of the non-promoter CTCF binding sites on day 0 overlapped those on day 8. What's more, a significant proportion of the non-promoter FAIRE peaks were cell type–specific (Figure 2A), implying the role of non-promoter regulatory elements in cell type–specific transcriptional regulation. We divided the non-promoter FAIRE peaks in day 0 and day 8 3T3-L1 cells into tertiles by FAIRE signal intensity, and defined adipocyte- or preadipocyte-specific FAIRE peaks as indicated by red or green boxes in the 4-by-4 table in Figure 2B. By this definition, we judged each non-promoter FAIRE peak as adipocyte-specific, preadipocyte-specific or invariant (Figure 2B). Figure 1, Figures S2 and S3 show examples of adipocyte-specific non-promoter FAIRE peaks (indicated by asterisks) in loci near *Klf15*, *Pparg*, *Cebpa* [16], [20], *Mgll* [36], *Srebf1* and *cidec* [37]—all of which are abundantly expressed in adipose tissue and induced during adipocyte differentiation (data not shown). Remarkably, multiple adipocyte-specific FAIRE peaks existed in the vicinity of these genes and included introns and downstream regions (Figure 1, Figures S2 and S3).

To determine whether non-promoter FAIRE peaks were functionally associated with cell type–specific gene expression, we analyzed the relationship between the presence of the adipocyte- or preadipocyte-specific non-promoter FAIRE peaks and the change in gene expression during adipocyte differentiation. Those FAIRE peaks were enriched in the vicinity of genes, expression levels of which were highly induced or suppressed during adipocyte differentiation (Figure 2C). Importantly, as the number of the adipocyte-specific FAIRE peaks associated with a gene increased, the fraction of up- or down-regulated genes increased or decreased, respectively (Figure 2D, red lines), while as the number of associated preadipocyte-specific FAIRE peaks increased, the fraction of up- or down-regulated genes decreased or increased, respectively (Figure 2D, green lines). Conversely, the more robust the induction of the expression level of a gene during adipocyte differentiation, the greater the numbers of adipocyte-specific FAIRE peaks associated with the gene (Figure 2E, red line). In contrast, the more robust the reduction of the expression levels of a gene during adipocyte differentiation, the greater the numbers of associated preadipocyte-specific FAIRE peaks that were associated (Figure 2E, green line). Invariant FAIRE peaks were associated specifically with neither up- nor down-regulated genes (Figure 2E, blue line). We next employed a gene ontology (GO) analysis tool (DAVID) [38] to determine what kind of biological processes were associated with genes bound by the adipocyte-specific FAIRE peaks. We found that biological processes (e.g., adipocyte differentiation) were significantly enriched compared with the genomic background (Figure 2F). It was of interest that embryonic placenta development—for which PPARγ is critical [13], [14], [15]—was enriched (Figure 2F). Together, these data highlight the role of the cell type–specific non-promoter open chromatin sites detected by FAIRE-seq in differentiation-dependent transcriptional regulation.

### Analysis of Binding Sites for PPARγ and RXRα in 3T3-L1 Adipocytes

PPARγ is key regulator of adipocyte development [16], [20]. To elucidate the contribution of PPARγ to adipocyte-specific transcriptional regulation, we conducted ChIP-seq analyses using antibodies specific for either PPARγ or RXRα [24] in 3T3-L1 adipocytes at 36 hours and day 8 after induction of differentiation. The number of PPARγ binding sites increased during differentiation while that of RXRα binding sites remained virtually constant (Figure 2G). Significant overlap between the PPARγ and RXRα binding sites was consistent with the heterodimer formation of PPARγ and RXRα [21], [39] (Figure 2G). Microarray and GO analysis revealed that the PPARγ binding sites were enriched in the vicinity of genes up-regulated by adipocyte differentiation (Figure S4B) and the bound genes were associated with adipocyte differentiation and lipid metabolism (Figure S4C). Using MEME [40], we performed de novo motif analysis of genomic regions bound by PPARγ, and found that the AGGTCA-n-AGGTCA (called DR-1) shown was the most over-represented one (E-value 1.3×10^−055^) (Figure S4A). An extension AGT 5′ outside of DR-1 appeared to correspond to the direct interaction between the DNA and the hinge region between the DNA-binding domain and the ligand-binding domain [41].

As shown in genomic loci (Figure 1, Figures S2 and S3), a significant proportion of adipocyte-specific non-promoter FAIRE peaks overlapped the PPARγ/RXRα binding sites. To determine the contribution of PPARγ to the adipocyte-specific open chromatin regions, we calculated percent fractions of the FAIRE peaks that were stratified by FAIRE signal in 3T3-L1 on day 0 and day 8 (Figure 2B) —and that overlapped either the PPARγ binding sites (Figure 2G) or C/EBPα binding sites in 3T3-L1 reported by Schmidt et al. [42]. Both PPARγ and C/EBPα binding sites were enriched in the fractions of adipocyte-specific FAIRE peaks (Figure 2H and 2I), and they respectively accounted for 45.3% and 11.7% of the adipocyte-specific FAIRE peaks (averages of the red bars in Figure 2H and 2I). These data support the role of PPARγ and C/EBPα as primary transcription factors that drive adipocyte-specific gene expression—although they may not explain all of it.

### Clustering of Multiple Adipocyte-Specific Non-Promoter FAIRE Peaks and the PPARγ Binding Sites

**Figure 3:**
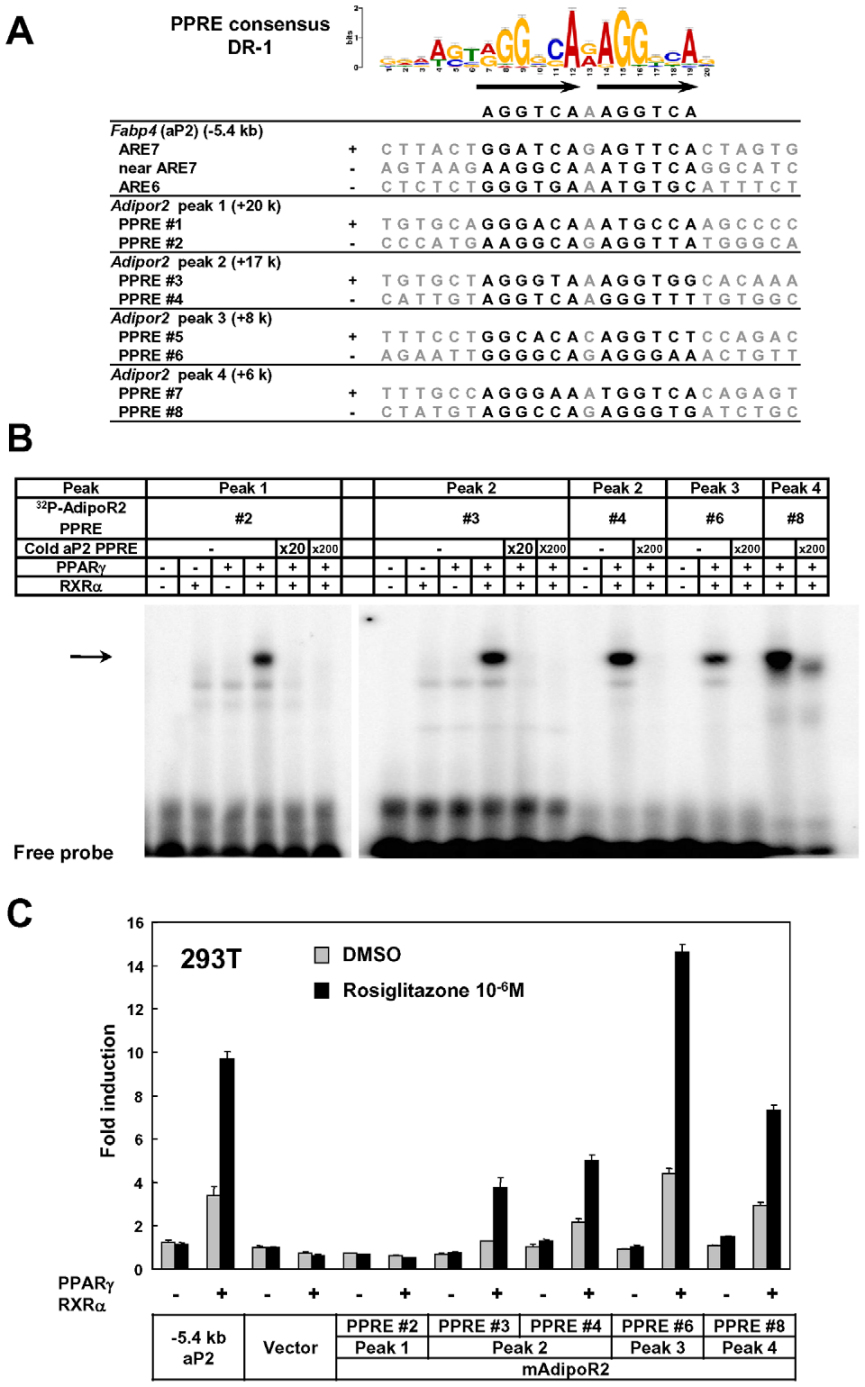
Identification of functional regulatory elements in the intron 1 of *Adipor2*. The PPARγ binding sites in the *Adipor2* gene locus (Figure S2B, arrow heads) were analyzed. (A) Putative DR-1 motifs (PPAR response elements or PPREs) in the regions. ARE6 and ARE7 in the −5.4 kb promoter upstream of *Fabp4*(aP2) were previously identified PPREs [87]. (B) Gel shift assay showing binding of the PPARγ/RXR heterodimer to the motifs. An arrow indicates the PPARγ/RXRα heterodimer bound by radiolabeled probe. Competition by cold oligos showed the specificity of the binding. (C) Luciferase reporter assay in HEK293T cells. Most of the motifs inserted into reporter vectors with the tk minimal promoter responded to over-expressed PPARγ/RXRα and stimulation with its synthetic ligand, rosiglitazone. The −5.4 kb promoter of PPARγ target gene *Fabp4* (aP2) [84] was included as a positive control.

Genes that were highly induced by adipocyte differentiation often harbored multiple adipocyte-specific FAIRE peaks and/or PPARγ binding sites in their vicinity, as suggested by the linear correlation between the number of the associated adipocyte-specific FAIRE peaks and the robustness of the induction of the gene by adipocyte differentiation (Figure 2D and 2E). (See Figure 1, Figures S2 and S3 for representative genes.) To determine whether these multiple regions have functional regulatory elements, we selected AdipoR2 [43], [44]. Although AdipoR2 was regulated by PPARγ and PPARα ([45] and data not shown), conventional −2 kb promoter studies failed to identify the response element [46]. Our ChIP-seq analysis revealed a cluster of multiple PPARγ/RXRα binding sites in the intron 1, downstream of the TSS of AdipoR2 (Figure S2B, arrow heads). We identified putative DR-1 motifs in these biding sites (Figure 3A) and tested them by gel-shift assay and luciferase assay. These motifs were indeed bound by the PPARγ/RXRα heterodimer, and were functional in the luciferase assay (Figure 3B and 3C), suggesting the functionality of these elements and the advantage of a genome-wide approach over the conventional “promoter-bashing” approach to identifying such response elements.

**Figure 4:**
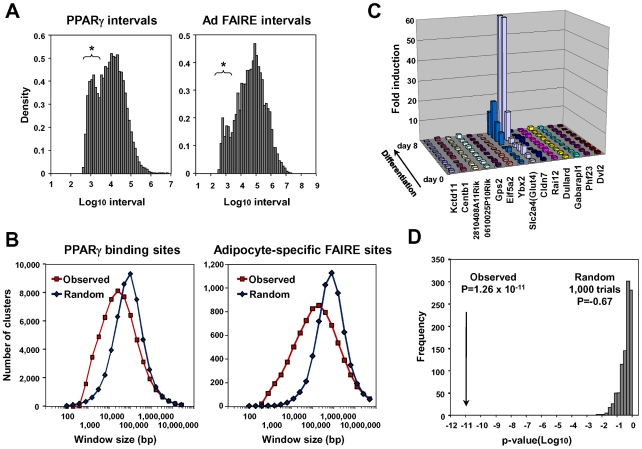
Statistical analyses for clustering of adipocyte-specific FAIRE peaks and PPARγ binding sites and co-regulation of neighbor genes during adipogenesis. (A) Histogram showing distribution of intervals (defined as distances to the nearest neighbor sites) among all PPARγ peaks (left) and among the adipocyte-specific FAIRE peaks (right). Note that there was increased occurrence of sites separated by short intervals (indicated by asterisks). See [48] for details of the method. (B) Clustering analysis of the PPARγ binding sites and the adipocyte-specific FAIRE peaks by counting the total number of clusters (defined as more than two peaks) determined for windows with indicated width. The PPARγ binding site or adipocyte-specific FAIRE peak clusters occurred more frequently in the observed data set than in random data with the same number of sites. The difference in the number of clusters was observed at window sizes ranging from 800 bp to 30∼100 kb compared with the random sample. See reference [47] for details of the method. (C) Microarray analysis showing both *Slc2a4 (Glut4)* and *Ybx2* included in the adipocyte-specific FAIRE peak cluster (Figure S2C) co-regulated during differentiation. (D) Neighbors of highly induced genes (>10 fold) were more likely to be up-regulated over three fold (18%, or 112 of 618 neighbors) than the 2,012 of 21,343 total genes (9%) that were up-regulated over three fold (p = 1.26×10^−12^, one-sided Fisher test). Neighbors of randomly selected genes were not significantly up-regulated (p = −0.67, average of 1,000 trials). See reference [50] for method.

Recent genome-wide studies revealed clustering of open chromatin regions detected by Dnase I hypersensitivity assay or by FAIRE in the genomes of CD4+ T cells [47], pancreatic islet cells [10], [48] and binding sites for certain transcription factors [49])—certainly the PPARγ binding sites and adipocyte-specific FAIRE peaks in our analyses tended to form clusters as indicated by an additional peak in distribution histograms of the distance to the nearest peak among the PPARγ binding sites or the adipocyte-specific FAIRE peaks (Figure 4A). We calculated the total number of PPARγ binding site clusters for different window sizes and compared them with a random data set comprised of the same number of sites (Figure 4B). The PPARγ binding sites had a significantly higher number of clusters in a window size raging from 800 bp to ∼30 kb. Similar results were obtained for the adipocyte-specific FAIRE peaks (Figure 4A and 4B).

On the other hand, multiple genes involved in adipocyte function [55], [56], [57] were often co-regulated in certain genomic regions that harbor clusters of adipocyte-specific regulatory elements (see Figure S2C, Figure 4C, and Figure S5). We therefore statistically tested—method in reference [50]—to see if neighboring genes tended to be co-regulated during adipocyte differentiation, and found that neighbors of highly induced genes (>10 fold) were indeed more likely to be up-regulated over three fold (18%, or 112 of 618 neighbors) than the 2,012 of 21,343 total genes (9%) that were up-regulated over three fold (P = 1.26×10^−12^, one-sided Fisher test). Neighbors of randomly selected genes were not significantly up-regulated (p = −0.67, average of 1,000 trials, Figure 4D). Together, these data suggest that the transcriptional regulation of genes during adipocyte differentiation involves multiple adipocyte-specific regulatory elements—which tend to form clusters—and that co-regulation of neighboring genes often occurs during adipocyte differentiation.

### Sequence Motif Analyses of DNA Sequences of the Adipocyte-Specific Non-Promoter FAIRE Peaks

**Figure 5:**
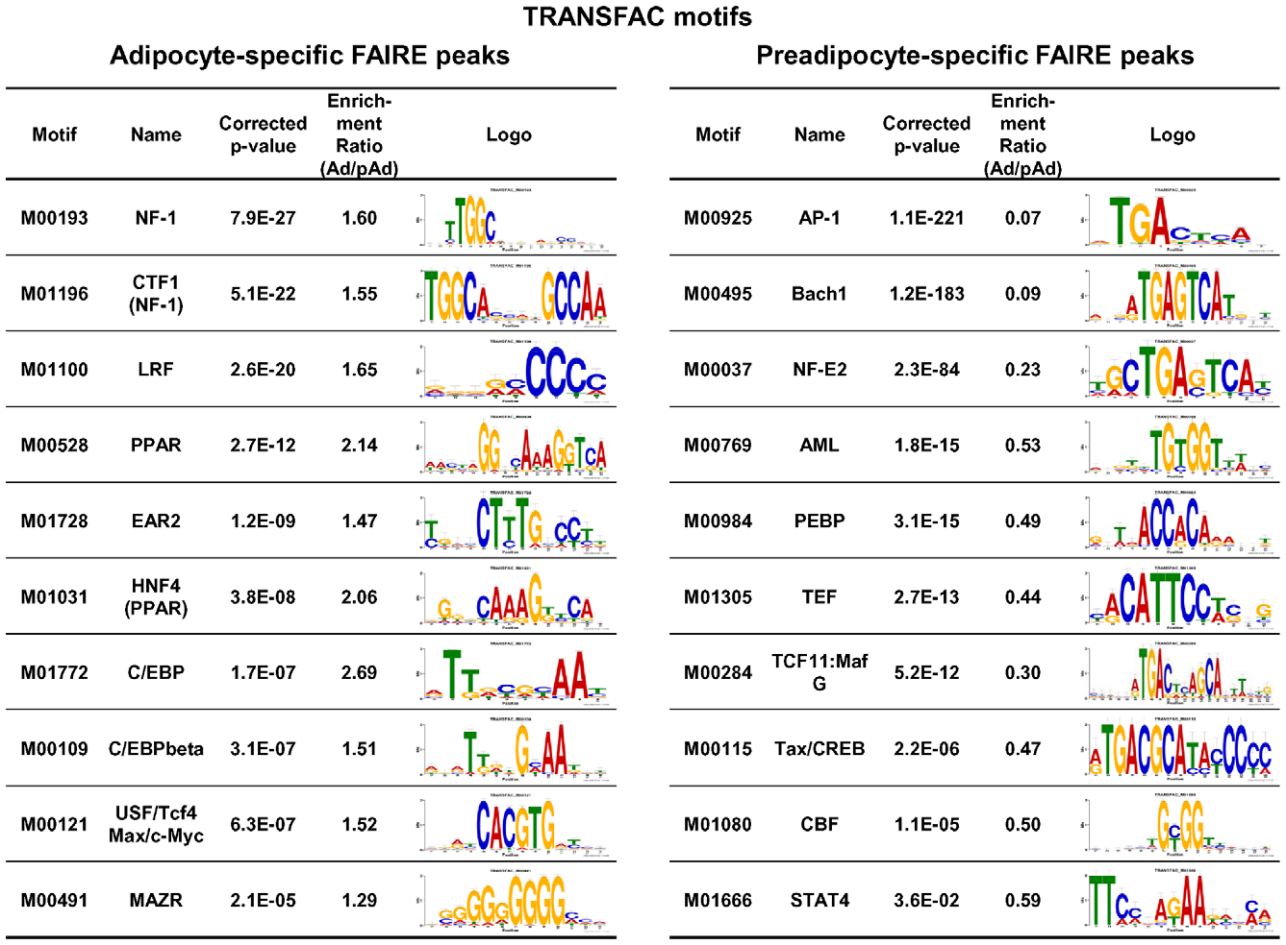
Known motif enrichment analysis of adipocyte- or preadipocyte-specific FAIRE peaks (TRANSFAC motifs). Enrichment analysis of the adipocyte- (left) and the preadipocyte-specific (right) FAIRE peaks for known motifs in the TRANSFAC database (Release 2010.4) performed by using AME in the MEME suite. After removing repeat regions with RepeatMasker [83], DNA sequences from the center 150 bp regions of the top 2,000 cell type–specific FAIRE peaks were analyzed (p-value report threshold : 0.05). Motif enrichment ratios (Ad/pAd FAIRE) for motifs in the TRANSFAC database were also determined by a method described in reference [28]. Motifs with an enrichment ratio greater than 1.20 (for the adipocyte-specific FAIRE peaks, left) or less than 0.833 (for the preadipocyte-specific FAIRE peaks, right) are shown in the table. See “Materials and Methods” for details.

Next, we performed enrichment analyses of known motifs using AME in the MEME suite and the TRANSFAC [51] and JASPER [52] motif databases to identify motifs enriched in either adipocyte- or preadipocyte-specific FAIRE peaks compared with the background (statistical values shown as corrected p-value in Figure 5). We also determined the enrichment ratio (Ad/pAd) by calculating the ratio of occurrence of a motif in the adipocyte-specific FAIRE peaks and in the preadipocyte-specific FAIRE peaks as described in reference [28]. Using both parameters, we obtained motifs that had been significantly enriched in either kind of FAIRE peak and that occurred in significantly different number. Figure 5 shows the top of the list of TRANSFAC motifs enriched in the adipocyte- and preadipocyte-specific FAIRE peaks. The motifs for PPARγ (and other DR1 motifs) and C/EBPs were among the list, consistent with their critical roles in adipogenic transcription. Motif analyses using the JASPER motif database showed enrichment of the motifs for PPARγ, C/EBPs and the motif for Zfp423, a recently identified adipogenic regulator [53] (Figure S6). Motif analyses of the preadipocyte-specific FAIRE peaks showed significant enrichment of a motif for AP-1, a downstream transcription factor complex of the growth factor/MAP kinase signaling pathways, which include epidermal growth factor and c-Jun N-terminal kinases, known inhibitors of adipogenesis [54], [55] (Figure 5 and Figure S6). We also performed de novo motif analysis (MEME) [40] of the adipocyte-specific FAIRE peaks, and observed significant enrichment of motifs that corresponded to those for PPARγ and C/EBPs (Figure S7). Together, these instances of enrichment of known regulators indicate the validity of this approach.

### Identification of NFI Family Transcription Factors as Novel Regulators of Adipocyte Differentiation

**Figure 6:**
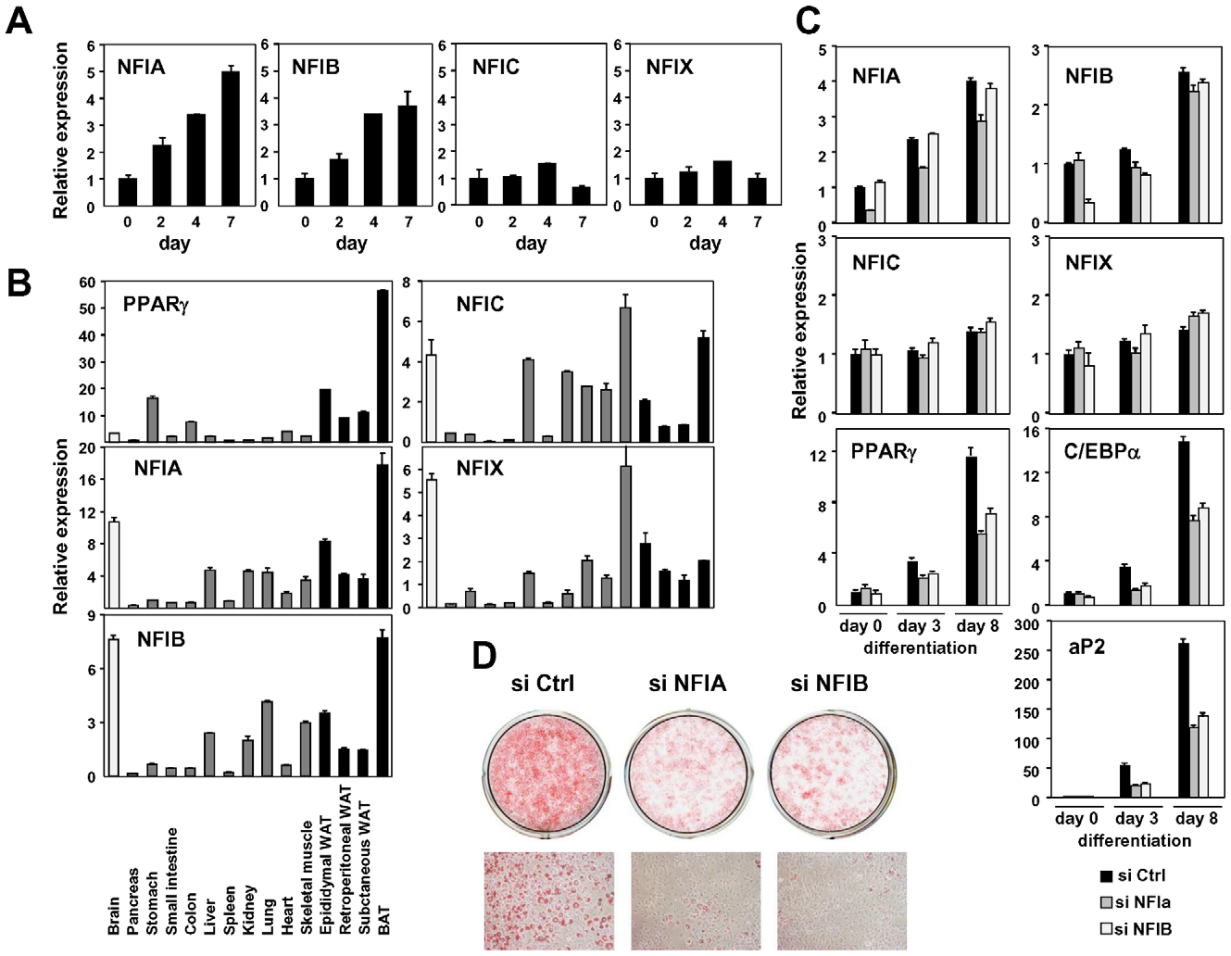
NFIA and NFIB are novel regulators of adipocyte differentiation. (A) Transcriptional regulation of NFI transcription factors during adipocyte differentiation (3T3-F442A). (B) Tissue distribution of the NFI family genes. Expression levels relative to 36B4 in various tissues were determined by qPCR. (C, D) Effects of siRNA-mediated knockdown of NFIA and NFIB on adipogenic gene expression (C) and lipid accumulation in 3T3-L1 adipocytes judged by oil red O staining (D). Knockdown of either NFIA or NFIB resulted in suppression of the induction of PPARγ, C/EBPα and the PPARγ target gene, aP2, as well as increase in lipid accumulation during adipocyte differentiation.

There were several other motifs for transcription factors, their functions not previously linked to adipocyte differentiation (Figure 5, Figures S6 and S7). We focused on a motif for the NFI family transcription factors. The murine NFI family consists of NFIA, NFIB, NFIC and NFIX, and was identified as a site-specific DNA-binding protein that bound to the adenovirus origin of replication [56]. It forms a dimer to bind to the symmetric consensus sequence TTGGC(N5)GCCAA [57]. We first examined the expression change of these factors in in vitro adipocyte differentiation and found that the expression of NFIA and NFIB were significantly induced during differentiation of 3T3-L1 and of another adipogenic cell line, 3T3-F442A (Figure 6A and 6C). Consistent with this pattern, both NFIA and NFIB were highly expressed in a variety of adipose tissue depots in addition to the brain (Figure 6B). We next examined the effect of siRNA knockdown of NFIA and NFIB on adipogenic gene regulation and adipocyte differentiation (Figure 6C). Interestingly, induction of the expression of the adipogenic transcription factors PPARγ and C/EBPα and of downstream genes was significantly suppressed by siRNA knockdown of either NFIA or NFIB (Figure 6C). Consistent with the gene expression change, we observed significant reduction of lipid accumulation as judged by oil red O staining, suggesting physiological roles for NFIA and NFIB in adipocyte differentiation (Figure 6D). We confirmed the effect of NFIA and NFIB knockdown on adipogenesis by using independent pooled siRNA (Figure S8).

**Figure 7:**
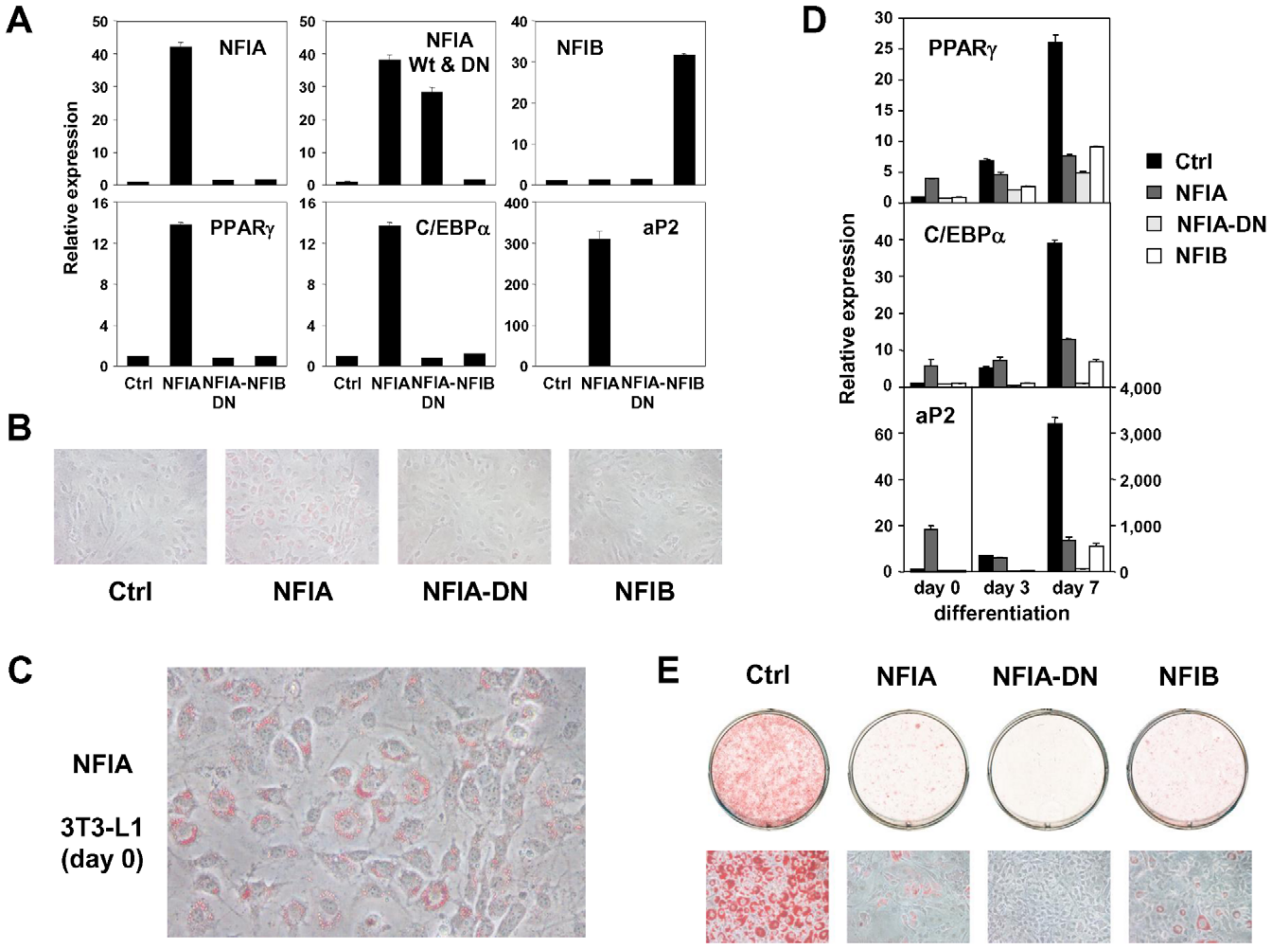
Overexpression of NFIA, NFIB, and dominant negative NFIA in 3T3-L1 cells. (A) Expression analysis of overexpressed NFI factors (upper panel) and adipogenic PPARγ, C/EBPα and aP2 (lower panel). Note, overexperssion of NFIA resulted in a robust induction of adipogenic factors. (B) Microscopic pictures of 3T3-L1 cells overexpressing NFI factors at confluence stained by oil red O (day 0). (C) Close examination of NFIA-overexpressing cells revealed formation of lipid droplets without adipogenic stimulus before differentiation. (D) Time course of expression levels of PPARγ, C/EBPα and aP2 during differentiation. Note, the induction of these genes by NFIA overexpression was overtaken by that of control cells, and on day 7, NFIA and NFIB overexpressing cells showed attenuated differentiation. Dominant negative NFIA showed almost complete suppression. (E) Oil red O staining of 3T3-L1 overexpressing NFI factors on day 7.

We next asked whether overexpression of these factors influence adipocyte differentiation. We amplified NFIA and NFIB coding sequences from cDNA prepared from adipocytes, and cloned them into retroviral pMXs-puro vectors. We also made a dominant negative NFIA that lacks the C-terminal transactivation/repression domain (NFIA-DN) [58]. Overexpression of NFIA—but not NFIA-DN or NFIB—resulted in robust induction of PPARγ, C/EBPα and aP2 (Figure 7A) at a basal state. Surprisingly, the induction of these factors was robust enough to make the cells to form lipid droplets visible and stainable by oil red O even before initiation of differentiation by the DMI (dexamethasone, IBMX and insulin) treatment (Figure 7B and 7C). However, after the DMI treatment, NFIA-expressing cells were overtaken by control cells, and on day 7, NFIA and NFIB overexpressing cells showed attenuated differentiation (Figure 7D and 7E). We speculate that this was caused by secondary effects of overly strong overexpression levels (>30 fold, Figure 7A). Almost complete suppression of adipogenesis by NFIA-DN overexpression was consistent with the results of knockdown experiments (Figure 6, Figure 7D and 7E). Nevertheless, the robust induction of PPARγ, C/EBPα and aP2 by NFIA overexpression at the basal state implies direct action of NFIA on transcriptional control of these factors.

**Figure 8:**
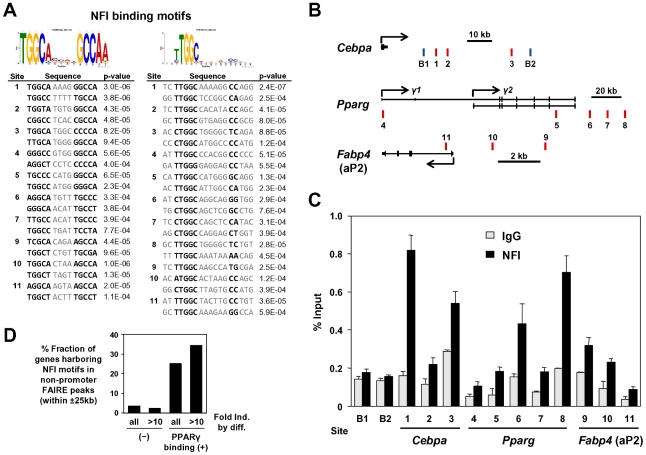
NFI occupy the adipocyte-specific FAIRE peaks and/or the PPARγ binding sites near PPARγ, C/EBPα, and aP2 genes. (A) The NFI binding motifs identified in the adipocyte-specific FAIRE peaks and/or the PPARγ binding sites in the vicinity of PPARγ, C/EBPα and aP2. For site numbers, see (B). (B) Genomic location of the regions examined. B1 and B2 are unrelated genomic regions used as background negative controls. (C) ChIP-qPCR analysis using an anti-NFI antibody (H-300). (D) Percent fraction of genes harboring NFI motifs in non-promoter FAIRE peaks (within ±25 kb) were higher when the genes were bound by PPARγ (within ±25 kb) and induced during differentiation.

To dissect the mechanism by which NFIs regulate PPARγ, C/EBPα and aP2, we examined DNA sequences of the adipocyte-specific FAIRE peaks and/or the PPARγ binding sites in the vicinity of these factors and found that some of them have NFI binding motifs as listed in Figure 8A. ChIP analysis using an anti-NFI antibody confirmed actual binding of NFI to these sites (Figure 8B and 8C). We extended this experiment by counting NFI motifs in the FAIRE peaks on a genome-wide scale. Interestingly, percent fractions of genes harboring NFI binding motifs in the FAIRE peaks were higher when the genes were bound by PPARγ and induced during differentiation (Figure 8D), indicating a significant degree of specificity for the NFI's action on the adipogenic transcriptional program.

Collectively, we demonstrated that the combination of FAIRE-seq and computational motif analyses is useful in identifying novel regulators of adipocyte differentiation.

### Comparison of FAIRE Peaks between Undifferentiated 3T3-L1 and NIH-3T3 Cells

The 3T3-L1 adipogenic cell line was established by isolating clonal sublines of mouse fibroblast line 3T3 [59]. Lastly, we compared FAIRE peaks between ‘undifferentiated’ 3T3-L1 and NIH-3T3 cells. As shown in Figure 2A, a substantial proportion of FAIRE peaks was unique to either 3T3-L1 or NIH-3T3 cells. We defined non-promoter FAIRE peaks as specific to 3T3-L1and NIH-3T3—as we did for the adipocyte- or preadipocyte-specific FAIRE peaks in Figure 2B. The 3T3-L1- or NIH-3T3-specific FAIRE peaks were enriched in the vicinity of genes whose expression levels were higher in 3T3-L1 or NIH-3T3, respectively (Figure S9A). Motif analysis of the 3T3-L1-specific FAIRE peaks showed that the binding motif for EBF (Figure S9B) had the highest enrichment ratio (1.81) and a statistically significant p-value of 3.9E-3. Although the p-value of the motif for PPARγ/RXR did not reach statistical significance, that motif had an enrichment ratio of 1.84. These two factors were among the handful that were proven to transform NIH-3T3 cells into adipocytes when ectopically introduced [16], [60].

## Discussion

We demonstrated that genome-wide mapping of open chromatin regions by FAIRE-seq is a simple, accurate method that allows a snapshot view of regulatory elements in the genome. Although open chromatin regions detected by FAIRE-seq include promoters of transcribed genes, enhancers and insulators, open chromatin regions that vary in two different conditions likely contain regulatory elements that play roles in the specific biological process. By comparing open chromatin regions in preadipocytes and adipocytes, we identified the adipocyte- and preadipocyte-specific FAIRE peaks in the genome. Functionally, we demonstrated that the adipocyte-specific FAIRE peaks were associated with genes up- regulated by adipogenesis while the preadipocyte-specific FAIRE peaks were associated with genes down-regulated by adipogenesis (Figure 2C, 2D and 2E). Adipocyte gene expression appears mediated through multiple regulatory elements distal to transcription start sites (TSSs): greater induction of gene expression by differentiation means greater likelihood that more adipocyte-specific FAIRE peaks are associated with the gene (Figure 2D and 2E). This implies that optimal gene transcriptional regulation may require coordinated actions of multiple regulatory elements. Therefore, although valuable and informative, the proximal promoter assay may not always be sufficient (e.g., AdipoR2, see Figure S2B and Figure 3). Nevertheless, the importance of proximal promoter regions is obvious given the fact that many proximal promoter regions are successfully used to generate tissue-specific transgenic lines. Recently, Mikkelsen et al. demonstrated in adipocytes that many *cis*-regulatory elements are often not conserved between human and murine adipocytes even though the expression pattern of genes is conserved [28]. They observed that such motifs were located within linage-specific transposon insertions. Existence of multiple regulatory elements around biologically important genes *could* be a mechanism by which cells maintain key gene regulations against genomic changes during evolution. Clustering of regulatory elements could also result from an accumulative effect of such evolutional genomic changes.

Computational motif analysis is used to discover new transcription-factor binding motifs in sequences inferred from genome-wide studies such as ChIP-seq [61]. In genome-wide ChIP analysis of transcription factors, motif analysis is used to obtain their accurate binding motifs *and* discover unknown DNA binding factors that co-localize with the transcription factors of interest, for example, see [27], [62], [63]. The analyses, however, relied on prior knowledge about transcription factors and the regions to be analyzed are limited to their biding sites. In contrast, the combination of motif analyses and mapping of regulatory elements by FAIRE-seq does not require such prior knowledge, hence offers a distinct advantage in unbiased screening for novel transcription factors important in given biological processes. In our study, we retrieved the motifs for PPARγ and C/EBPs and for known regulators that top the list of the motifs identified in the adipocyte- or preadipocyte-specific FAIRE peaks (Figure 5, Figures S6 and S7). Furthermore, we demonstrated that NFIA and NFIB were functionally required for proper adipocyte differentiation (Figure 6). These results demonstrated that motif analyses of cell type–specific FAIRE peaks are useful in identifying regulators of a biological process in an unbiased manner.

To our knowledge, few studies have employed motif analysis and our unbiased approaches in investigating enhancer-like DNA regions. Mikkelsen et al. recently employed ChIP-seq for H3K27ac to define enhancer regions specific for adipocyte differentiation. Both studies similarly detected the motifs for PPAR, C/EBPs and AP-1 in the most enriched motifs. There are, however, differences. Mikkelsen discovered PLZF and SRF as novel negative regulators [28] and we found NFIA and NFIB as regulators of adipocyte differentiation—perhaps due to differences in methods. First, we directly compared FAIRE peaks and H3K27ac peaks detected in the Mikkelsen study and found considerable, but not complete, overlap especially in the non-promoter regions: 94% of 10,461 promoter FAIRE peaks and 45% of 27,320 non-promoter FAIRE peaks overlapped H3K27ac in 3T3-L1 on day 0. There may be different classes of enhancer elements that prefer either H3K27ac or open chromatin. Also, we used two parameters to sort motifs: the statistical significance of enrichment (p-value) in either kind of cell type–specific FAIRE peaks; and the motif enrichment ratio between the adipocyte- and preadipocyte-specific FAIRE peaks (see [28]). The combination guarantees significant enrichment of the peaks' motifs and the difference in their number depending on whether they are adipocyte- or preadiocyle-specific. The motifs for PLZF and SRF were not on the top of our list since the p-values were not significant—probably due to relatively lower occurrence, although we also found a significant enrichment ratio of 0.37 and 0.50, respectively. We calculated p-values and the enrichment ratios of the top motifs in the Mikkelsen's study by using our adipocyte- and preadipocyte-specific FAIRE peaks and found general similarity (Figure S10). Overall, both studies notably demonstrate the utility of the combining computational motif analysis and unbiased mapping of regulatory elements in identifying new regulators of adipocyte differentiation.

Siersbæk et al. recently employed DNase-seq to investigate genome-wide change in open chromatin structure at various time points during 3T3-L1 differentiation [6]. They reported dramatic increase in the number of open chromatin sites in the first hours of differentiation. Such regions included what they termed “hot spots” that were bound by multiple adipogenic regulators, facilitating binding of PPARγ and C/EBPα during the late stage of differentiation. We found that the DNaseI hypersensitive sites in 3T3-L1 cells on day 0 or day 6 in the Siersbaek study [6] significantly overlapped the FAIRE peaks on day 0 or day 8 in our study (78.8% and 80.9%, respectively) (Figure S11), suggesting that both methods detect similar open chromatin regions. Although limited amount of motif analyses of the DNase I sites was conducted in their study, we think a combination of motif analysis and DNase-seq should work in a similar way.

The NFI family was identified as site-specific DNA-binding protein that bound to the adenovirus origin of replication [56], [57]. Although defects in development of organs such as brain, lung, tooth, bone and skeletal muscle in *Nfia*, *Nifb*, *Nifc* and *Nfix*-deficient mice were documented [64], [65], [66], [67], [68], [69], no publication has reported direct evidence that NFI family transcription factors are involved in adipogenesis, but it is a reasonable supposition since bone, muscle and adipocytes have a common mesenchymal precursor [70]. Interestingly, Graves et al. demonstrated that NFI was bound to the adipogenic −5.4 kb enhancer region in the aP2 promoter [71], which is the original adipogenic enhancer region where the PPARγ/RXR heterodimer was found to bind and act [72]. The NFI binding motif they examined by gel shift assay [72] was close to the best-characterized PPARγ binding sites in the region, and was also in site 9 (Figure 8A, right panel, site 9), which was indeed bound by NFI in ChIP assay (Figure 8C). Forced overexpression of NFIA in 3T3-L1 cells dramatically induced expression of PPARγ, C/EBPα and aP2 and caused lipid droplet formation before initiation of differentiation. Our ChIP data suggest that activation of these genes by NFIA is through direct binding of NFI to regulatory elements near these genes. In overexpression experiments, NFIB did not activate the adipogenic genes (Figure 7). NFI factors are known to undergo extensive alternative splicing [57]. We speculate that this could be due to truncation of the C-terminus caused by lack of exons 10 and 11 in the NFIB cDNA that we cloned (NM_001113209.1) while the NFIA clone completely matched NM_010905.3. NFI was also implicated in functions of other nuclear receptors such as the androgen receptor (AR), estrogen receptor (ER) and glucocorticoid receptor [4], [73], [74]. Further studies are necessary to elucidate the mode of action of NFIs and positioning of NFIs in the adipogenic regulatory network.

## Materials and Methods

### Cell Culture

3T3-L1, NIH-3T3, 3T3-F442A and HEK293T cells were maintained in DMEM, supplemented with 10% FBS. For adipocyte differentiation, two days after confluence, 3T3-L1 cells were treated with dexamethasone (1 µM), IBMX (0.5 mM), and insulin (5 µg/ml) (DMI) for 48 hours, followed by treatment with insulin alone, with medium replacement every two days thereafter. For differentiation of 3T3-F442A, cells were treated with insulin (5 µg/ml) after confluence, with medium replacement every two days.

### Animal Studies

All animal works have been conducted according to the institutional guidelines.

### Antibodies

Generation of characterization of antibodies for human PPARγ and human RXRα was described previously [24]. Rabbit polyclonal anti-histone H3 trimethyl K4 (ab8580) was from Abcam. Antibodies against CTCF were from Upstate (#07–729). Anti-NFI antibody (H-300) was from Santa Cruz (sc-5567).

### FAIRE

FAIRE experiments were performed based on a protocol published by Giresi et al. [7]. Briefly, cells were fixed with 1% formaldehyde for five minutes at room temperature, the fixation stopped by adding 2.5 M glycine (final 125 mM). Fixed cells were scraped and collected in 15 ml tubes (4×10∧6 cells/tube) and washed twice with cold PBS, then 8×10^6^ cells were re-suspended in 800 µl of MC lysis buffer (10 mM Tris-HCl pH 7.5, 10 mM NaCl, 3 mM MgCl_2_, 0.5% NP-40) and incubated on ice for ten minutes. After spinning for four minutes at 8000 rpm, the pellet was re-suspended in 400 µl SDS lysis buffer (1% SDS, 10 mM EDTA, 50 mM Tris-HCl pH 8.0, proteinase inhibitor cocktail) and incubated on ice for ten minutes. Glass beads (size, 200 mg) (Polysciences Inc. #05483-250) were added and the DNA was sheared by sonicator. Next, we added 200 µl cold ChIP dilution buffer (0.01% SDS, 1.1% Triton X-100, 1.2 mM EDTA, 16.7 mM Tris-HCl pH 8.0, 167 mM NaCl), and after spinning for one minute at 8,000 rpm, supernatant was transferred to a new 1.5 ml tube. Aliquote was taken, de-crosslinked, purified by phenol/chloroform extraction, and run on a gel to ensure average fragment sizes of 300 bp. Remaining samples were processed three times by phenol/chloroform extraction to recover DNA not bound by nucleosome in the water phase. The samples were de-crosslinked by overnight incubation at 65°C and purified by ethanol precipitation. They were subsequently treated with RNase A (final 50 ug/ml), purified by QIAquick PCR purification kit (Qiagen) and used for subsequent analyses.

### Chromatin Immunoprecipitation (ChIP)

ChIP was performed as descried previously [24], [75]. For ChIP using anti-PPARγ, RXRα and CTCF antibodies, 3T3-L1 cells were cross-linked with 1% formaldehyde for ten minutes at room temperature and were prepared for ChIP as described previously. For ChIP using anti-H3K4me3 antibody, the nuclei of 3T3-L1 cells were prepared by centrifugation through a sucrose gradient and were digested with MNase (TaKaRa). After centrifugation, the supernatant was used for ChIP. Sequences of primers used for qPCR were listed in Table S1.

### High-Throughput Sequencing and Peak Detection

High-throughput sequencing was performed by using the Genome Analyzer System (GA II) (Illumina) as described elsewhere [76]. In short, we repaired ends of DNA samples, created 3′-dA overhang, ligated Illumina adaptors, size-fractioned the samples by gel extraction and amplified them with 8 cycles of PCR according to the manufacturer's instructions. We then purified the DNA and performed cluster generation and 36 cycles of sequencing on an Illumina cluster station and 1G analyzer following the manufacturer's instructions. Sequences were mapped to the reference murine genome, NCBI build 37 (mm9). Peak detection was performed using Findpeaks 3.1.9.2 [77] with a false discovery rate (FDR) cut-off of 1×10^−4^. Operations such as intersections, unions, and subtractions of genome regions were performed with a web-based GALAXY genome analysis tool [78], [79].

### Average Signal Profiling

Average profiling of FAIRE and histone modifications near transcription start sites or FAIRE peaks were generated using “sitepro” in the CEAS package [80].

### Adipocyte- and Preadipocyte-Specific FAIRE Peaks

For definition, we first ranked peaks based on signal intensity, which were detected in 3T3-L1 on either day 0 or day 8 with a FDR of 10^−4^. We then classified each peak into tertiles (high, mid, low) for either day the peak that had the higher percentile (see also the 4-by-4 table in Figure 3B).

### Gene Ontology Analysis

Gene ontology annotation analysis was performed using DAVID (ver. 6.7) [38]. The top 2,000 genes were used, sorted by the number and maximum height of the adipocyte-specific FAIRE peaks within a region ±25 kb from TSS. For genes bound by PPARγ, we used the top 931 genes with more than three PPARγ binding sites within a region ±25 kb from TSS. To detect enrichment of specific—rather than general—terms, following the instructions of DAVID's developer, we used GOTERM_BP_4 and GOTERM_BP_5, and sorted result lists by using both fold enrichment and Benjaini p-value [38], [81].

### Clustering Analysis

Statistical clustering analyses of the PPARγ binding sites and the adipocyte-specific FAIRE peaks were performed as described in references [47], [48].

### Enriched Motif Analysis

Enrichment analyses of known motifs were performed with AME ver. 4.6.0 in the MEME suite [82]. After removing repeat regions with RepeatMasker [83], DNA sequences from the center 150 bp regions of the top 2,000 cell type–specific FAIRE peaks were analyzed with a fixing partition of 2,000, dinucleotide randomization and p-value threshold of 10^−4^ and p-value report threshold of 0.05. We used the licensed version of TRANSFAC database (Release 2010.4) [51] and the JASPAR CORE database [52].

Motif enrichment ratios (adipocyte-/preadipocytes-specific FAIRE) for motifs in the TRANSFAC or JASPAR CORE database were determined by a method described in reference [28]. Instances of motifs were enumerated in the adipocyte- or preadipocytes-specific FAIRE peaks by using FIMO ver. 4.6.0 in the MEME suite, with a p-value threshold of 10^−4^, normalized by total nucleotide length. Motif enrichment ratios were determined by dividing the normalized adipocyte enrichment values by preadipocyte values.

MEME ver. 4.3.0 [40] was used to identify de novo motifs over-represented in the adipocyte- or preadipocyte-specific FAIRE peaks and the PPARγ binding sites. After removing repeat regions with RepeatMasker [83], DNA sequences from the center 150 bp regions of the top 800 cell type–specific FAIRE peaks with higher signals were used for the analyses. Identified enriched de novo motifs were next analyzed by TOMTOM in the MEME suite for comparison against a database of known motifs.

### Gel Shift Assay and Reporter Assay

The Gel shift assay and luciferase reporter assay were performed as previously described [84], [85]. For the luciferase assay, putative PPRE motifs were cloned in tandem (3× or 6×) into pGL3 basic reporter plasmid (Promega) together with the tk minimal promoter. The −5.4 kb aP2 promoter luciferase construct is described in reference [84].

### Knockdown of NFIA and NFIB by siRNA in 3T3-L1 Cell Differentiation

The 3T3-L1 cells were transfected with either control siRNA or siRNA for murine NFIA and NFIB (Santa Cruz Biotechnology, sc-37007, sc-36045 and sc-43566, Sigma MISSION siRNA, SASI_Mm02_00309629, 00309630, 00307243, 00307244) by using Lipofectamine RNAiMAX (Invitrogen) just before they reached confluence. Induction of differentiation (the DMI treatment) was started two days after confluence, as described in a method for differentiation of 3T3-L1 cells.

### Oil-Red-O Staining

The 3T3-L1 adipocytes were washed with PBS, fixed with formalin for 30 minutes at room temperature, rinsed with 60% isopropanol and stained with oil red O solution—freshly made by mixing 0.5% oil red O in isopropyl alcohol and water (3∶2)—and left to sit for one hour; the cells were then washed with water and dried.

### mRNA Expression Analysis

Total RNA was isolated using TRIzol reagent (Invitrogen), then 0.5 µg of the total RNA was reverse transcribed using high-capacity cDNA reverse transcription kits (Applied Biosystems #4375222) and random hexamers. Real-time quantitative PCR (SYBR green) analysis was performed on a 7900HT Fast Real-Time PCR System (Applied Biosystems). Primer sequences are listed in Table S1. Expression was normalized to 36B4.

### Microarray Analysis

Transcriptome analysis of 3T3-L1 during differentiation by using a GeneChip Mouse Genome 430 2.0 array (Affimetrix) was described previously [24]. Heat maps were generated by using GENOMICA, developed by Yaniv Lubling and Eran Segal at the Weizmann Institute of Science. Microarray data of 3T3-L1 and NIH-3T3 cells used in Figure S11 was obtained from GEO (accession number GSE10246).

### Retroviral Expression System

We amplified NFIA and NFIB coding sequences from cDNA prepared from adipocytes using primers listed in Table S1, and cloned them into retroviral pMXs-puro vectors. We also made a dominant negative NFIA that lacks the C-terminal transactivation/repression domain (NFIA-DN) [58]. Plat E cells were transfected with pMXs-puro plasmids using Lipofectamine 2000 (Invitrogen). Culture medium containing viruses after two day incubation was centrifuged at 2,000 rpm for 5 min and supernatant was collected and supplemented with 10 µg/ml polybrene. Conditioned medium with viruses was used to infect 3T3-L1 cells and then selection was started by adding 2 µg/ml puromycin and incubated for 2 days.

### Accession Numbers

FAIRE-seq and ChIP-seq raw data are deposited into the DNA data bank of Japan (DDBJ accession number: DRA000378).

## Supporting Information

Figure S1 Genomic distribution and characterization of promoter and non-promoter FAIRE peaks in 3T3-L1. (A) Location analysis of FAIRE peaks relative to RefSeq genes in 3T3-L1 (day 0). Promoter FAIRE peaks were defined as those located within +/−500 bp of RefSeq transcription start sites (TSSs). Notably, only 8% of the non-promoter FAIRE peaks were located in the −5 kb proximal promoter region, and the vast majority of them were located in distal regions such as introns and intergenic regions. (B) Average profiles of FAIRE and H3K4me3 signals around the TSSs of genes with high, moderate and low expression levels. Signal intensity from microarray data was used for classification by the signal's expression levels. The X-axis indicates distance from the TSS. (C) Percent fractions of the FAIRE peaks (promoter and non-promoter) that overlapped CTCF binding sites as well as H3K4me1 and H3K4me3 positive regions. (D) Average profiles of FAIRE, H3K4me1, H3K4me3, and H3K27ac signals around the FAIRE peaks in promoter and non-promoter regions. The X-axis shows distance from the center of the FAIRE peaks. The FAIRE peaks located within +/−100 bp from RefSeq TSSs were analyzed for promoter FAIRE peaks. The promoter FAIRE peaks showed H3K4me3(+)/H3K4me1(−) modification whereas the non-promoter FAIRE peaks showed H3K4me3(−)/H3K4me1(+) modification.
(TIF)

Figure S2 Clustering of multiple adipocyte-specific non-promoter FAIRE peaks and PPARγ binding sites near *Mgll*, *Adipor2* and *Slc2a4*. Clusters of multiple adipocyte-specific FAIRE peaks and/or PPARγ binding sites were located in genomic regions near *Mgll* (A), *Adipor2* (B) and *Slc2a4*(Glut4) (C) in 3T3-L1 adipocytes. In some cases—e.g., *Slc2ar4* (Glut4) and *Ybx2* in (C)—multiple genes were located in such regions. Bars below the FAIRE signal represent statistically significant FAIRE positive peaks (FDR<10^−4^). Red asterisks indicate the adipocyte-specific FAIRE peaks on day 8 (see Figure 2B for definition). Blue arrow heads in (B) indicate the PPARγ binding regions in the intron 1 of *Adipor2* tested in Figure 3.
(TIF)

Figure S3 Clustering of multiple adipocyte-specific non-promoter FAIRE peaks and PPARγ binding sites near *Cebpa*, *Srebf1*and *Cidec*. Clusters of multiple adipocyte-specific FAIRE peaks and/or PPARγ binding sites were located in genomic regions near *Cebpa* (A), *Srebf1* (B) and *Cidec* (C).
(TIF)

Figure S4 Binding sites for PPARγ and RXRα in 3T3-L1 cells. (A) De novo motif analysis (MEME) of the center 150 bp of the PPARγ/RXRα binding regions (top 400) in 3T3-L1, day 8. Of note, there is a 5′ extension AGT, which corresponds to the interaction between the PPARγ hinge region and DNA identified by crystal structure analysis [41]. (B) A heat map showing enrichment of PPARγ in the vicinity of genes up-regulated during differentiation. The horizontal bars in the right panel indicate each gene bound by PPARγ (+/−25 kb from TSS, day 8) (C) Ontology analysis with DAVID of genes bound by PPARγ [13].
(TIF)

Figure S5 Co-regulation of neighboring genes during adipocyte differentiation. (A, B) Genomic loci near (A) co-regulated *Mrpl12*, *Slc25a10* and *Gcgr* and (B) co-regulated *Hsd11b1*, *G0s2* and *Lamb3*. Note, there are clusters of the adipocyte-specific FAIRE peaks (asterisks) and the PPARγ binding sites encompassing the co-regulated genes. (C) Microarray analysis showing co-regulation of *Mrpl12*, *Slc25a10* and *Gcgr*, and co-regulation of *Hsd11b1*, *G0s2* and *Lamb3* included in the clusters of multiple adipocyte-specific FAIRE peak and PPARγ binding sites.
(TIF)

Figure S6 Known motif enrichment analysis of the adipocyte- or preadipocyte-specific FAIRE peaks (JASPAR CORE motifs). Enrichment analysis of the adipocyte- (left) and the preadipocyte-specific (right) FAIRE peaks for known motifs in the JASPAR CORE database performed with AME in the MEME suite by the same methods used in Figure 5.
(TIF)

Figure S7 De novo motif analysis of the adipocyte-specific FAIRE peaks. MEME ver. 4.3.0 was used to identify de novo motifs over-represented in the adipocyte- and preadipocyte-specific FAIRE peaks and PPARγ binding sites. After removing repeat regions, DNA sequences from the center 150 bp regions of top 800 cell type–specific FAIRE peaks with higher signals were used for the analyses. Identified enriched de novo motifs were analyzed by TOMTOM in the MEME suite for comparison against a database of known motifs.
(TIF)

Figure S8 Suppression of adipocyte differentiation by knockdown of NFIA and NFIB by using different siRNAs.
(TIF)

Figure S9 Comparison of FAIRE Peaks between undifferentiated 3T3-L1 and NIH-3T3 cells. (A) A heat map showing enrichment of the 3T3-L1- and NIH-3T3-specific FAIRE peaks in the vicinity (+/−25 kb from TSS) of genes sorted by using the ratio of expression levels in 3T3-L1 or NIH-3T3. The FAIRE peaks specific to 3T3-L1 or NIH-3T3 were enriched in the vicinity of genes whose expression levels were higher in 3T3-L1 or NIH-3T3, respectively. (B) Known motif analysis of the 3T3-L1-specific FAIRE peaks (vs NIH-3T3). The binding motif for EBF and PPARγ/RXR were among the top scored motifs.
(TIF)

Figure S10 The enrichment ratios of the top motifs in Mikkelsen's study [28] by using the adipocyte- and preadipocyte-specific FAIRE peaks.
(TIF)

Figure S11 Comparison of DNase-seq in Siersbæk's study [6] and FAIRE-seq peaks near *Klf5*, *Pparg* and *Cebpa* gene. DHS stands for DNase I hypersensitive sites.
(TIF)

Table S1 Sequences of primers.
(DOC)
