# Supplementary figures and images for: Global Mapping of Cell Type–Specific Open Chromatin by FAIRE-seq Reveals the Regulatory Role of the NFI Family in Adipocyte Differentiation
Source: PLoS Genet. 2011 Oct 20;7(10):e1002311. doi: 10.1371/journal.pgen.1002311 (PMC3197683; doi:10.1371/journal.pgen.1002311)

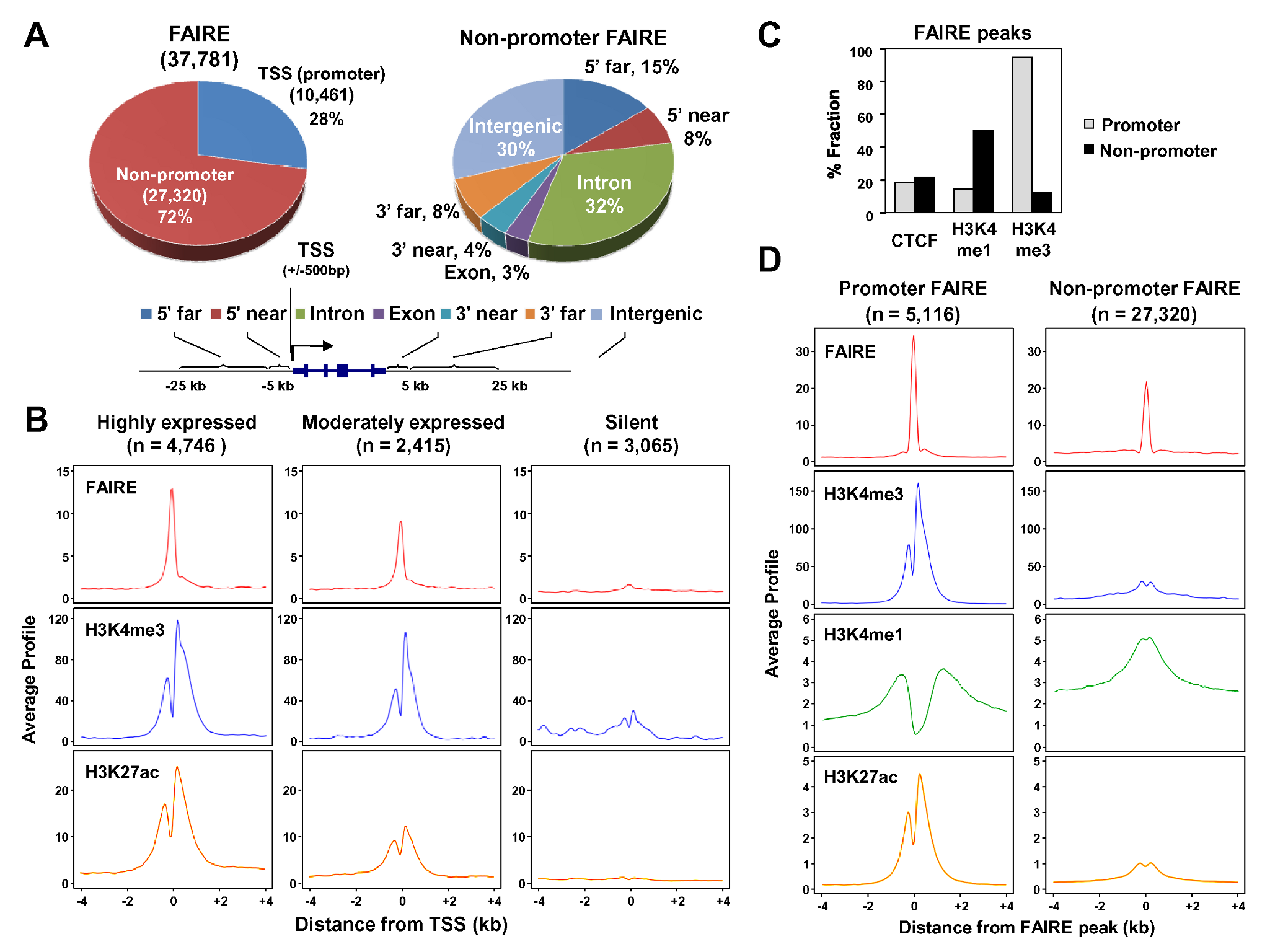

Supplement: Figure S1 — Genomic distribution and characterization of promoter and non-promoter FAIRE peaks in 3T3-L1. (A) Location analysis of FAIRE peaks relative to RefSeq genes in 3T3-L1 (day 0). Promoter FAIRE peaks were defined as those located within +/−500 bp of RefSeq transcription start sites (TSSs). Notably, only 8% of the non-promoter FAIRE peaks were located in the −5 kb proximal promoter region, and the vast majority of them were located in distal regions such as introns and intergenic regions. (B) Average profiles of FAIRE and H3K4me3 signals around the TSSs of genes with high, moderate and low expression levels. Signal intensity from microarray data was used for classification by the signal's expression levels. The X-axis indicates distance from the TSS. (C) Percent fractions of the FAIRE peaks (promoter and non-promoter) that overlapped CTCF binding sites as well as H3K4me1 and H3K4me3 positive regions. (D) Average profiles of FAIRE, H3K4me1, H3K4me3, and H3K27ac signals around the FAIRE peaks in promoter and non-promoter regions. The X-axis shows distance from the center of the FAIRE peaks. The FAIRE peaks located within +/−100 bp from RefSeq TSSs were analyzed for promoter FAIRE peaks. The promoter FAIRE peaks showed H3K4me3(+)/H3K4me1(−) modification whereas the non-promoter FAIRE peaks showed H3K4me3(−)/H3K4me1(+) modification. (TIF) [file pgen.1002311.s001.tif]

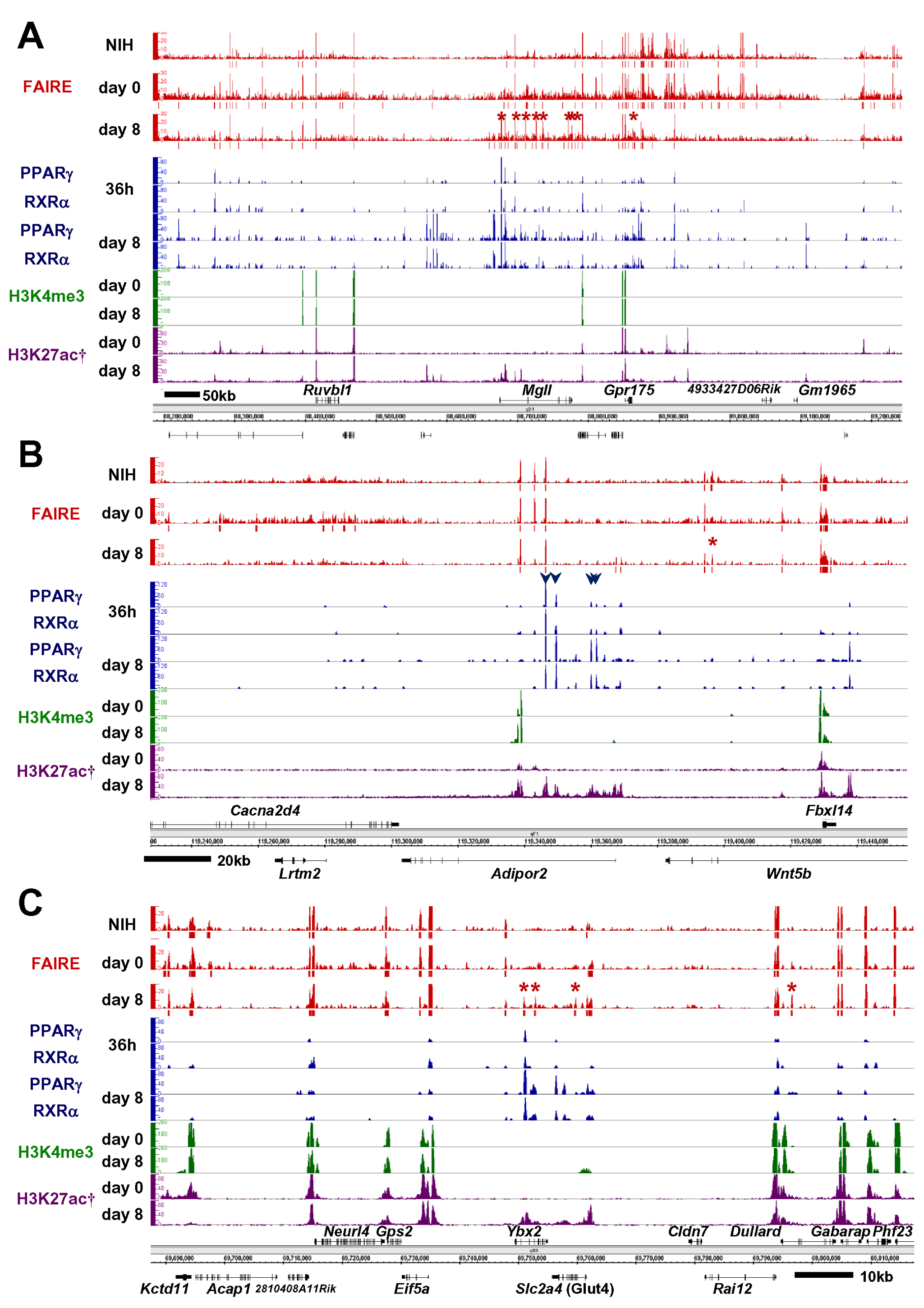

Supplement: Figure S2 — Clustering of multiple adipocyte-specific non-promoter FAIRE peaks and PPARγ binding sites near Mgll, Adipor2 and Slc2a4. Clusters of multiple adipocyte-specific FAIRE peaks and/or PPARγ binding sites were located in genomic regions near Mgll (A), Adipor2 (B) and Slc2a4(Glut4) (C) in 3T3-L1 adipocytes. In some cases—e.g., Slc2ar4 (Glut4) and Ybx2 in (C)—multiple genes were located in such regions. Bars below the FAIRE signal represent statistically significant FAIRE positive peaks (FDR<10−4). Red asterisks indicate the adipocyte-specific FAIRE peaks on day 8 (see Figure 2B for definition). Blue arrow heads in (B) indicate the PPARγ binding regions in the intron 1 of Adipor2 tested in Figure 3. (TIF) [file pgen.1002311.s002.tif]

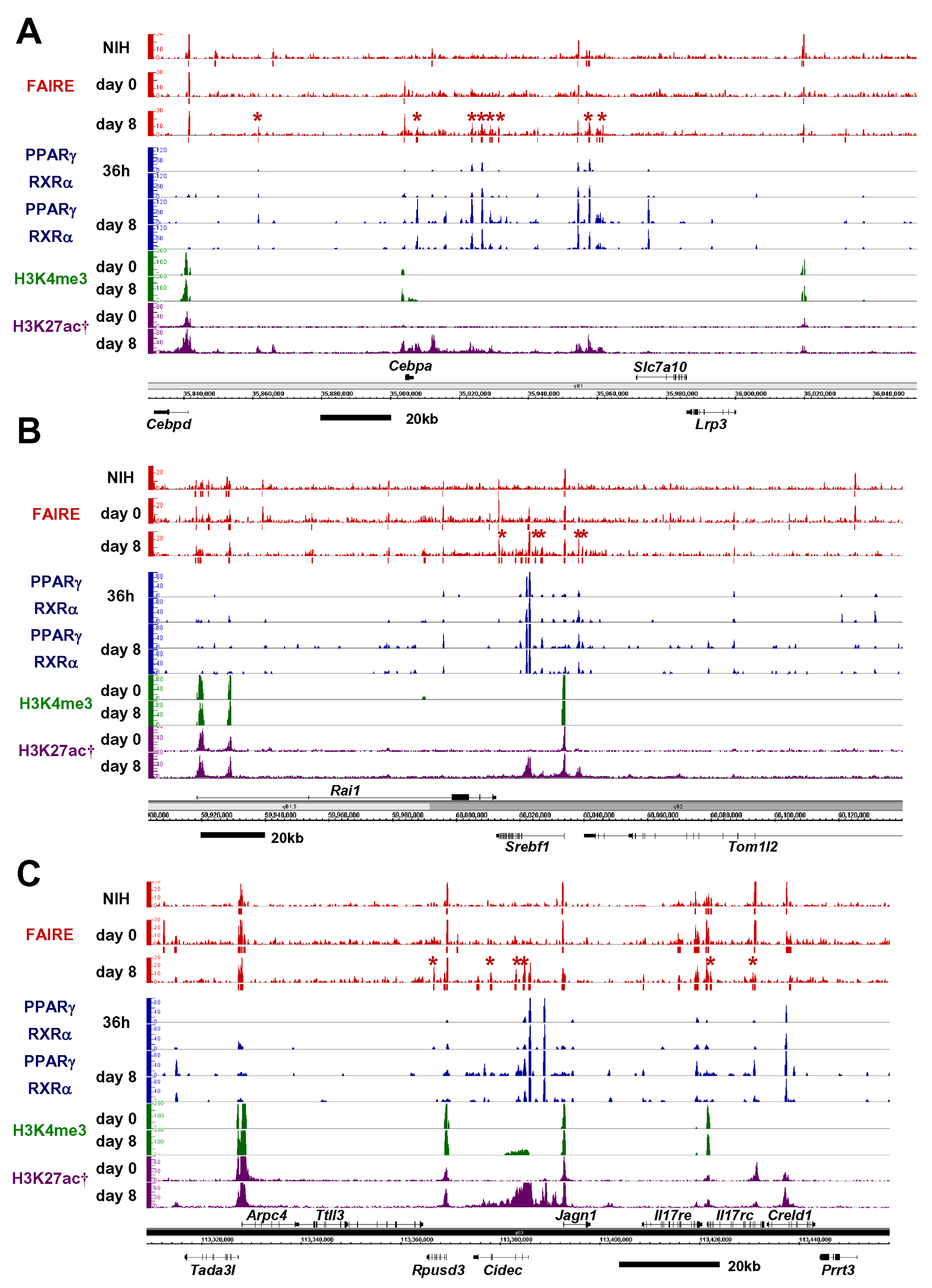

Supplement: Figure S3 — Clustering of multiple adipocyte-specific non-promoter FAIRE peaks and PPARγ binding sites near Cebpa, Srebf1and Cidec. Clusters of multiple adipocyte-specific FAIRE peaks and/or PPARγ binding sites were located in genomic regions near Cebpa (A), Srebf1 (B) and Cidec (C). (TIF) [file pgen.1002311.s003.tif]

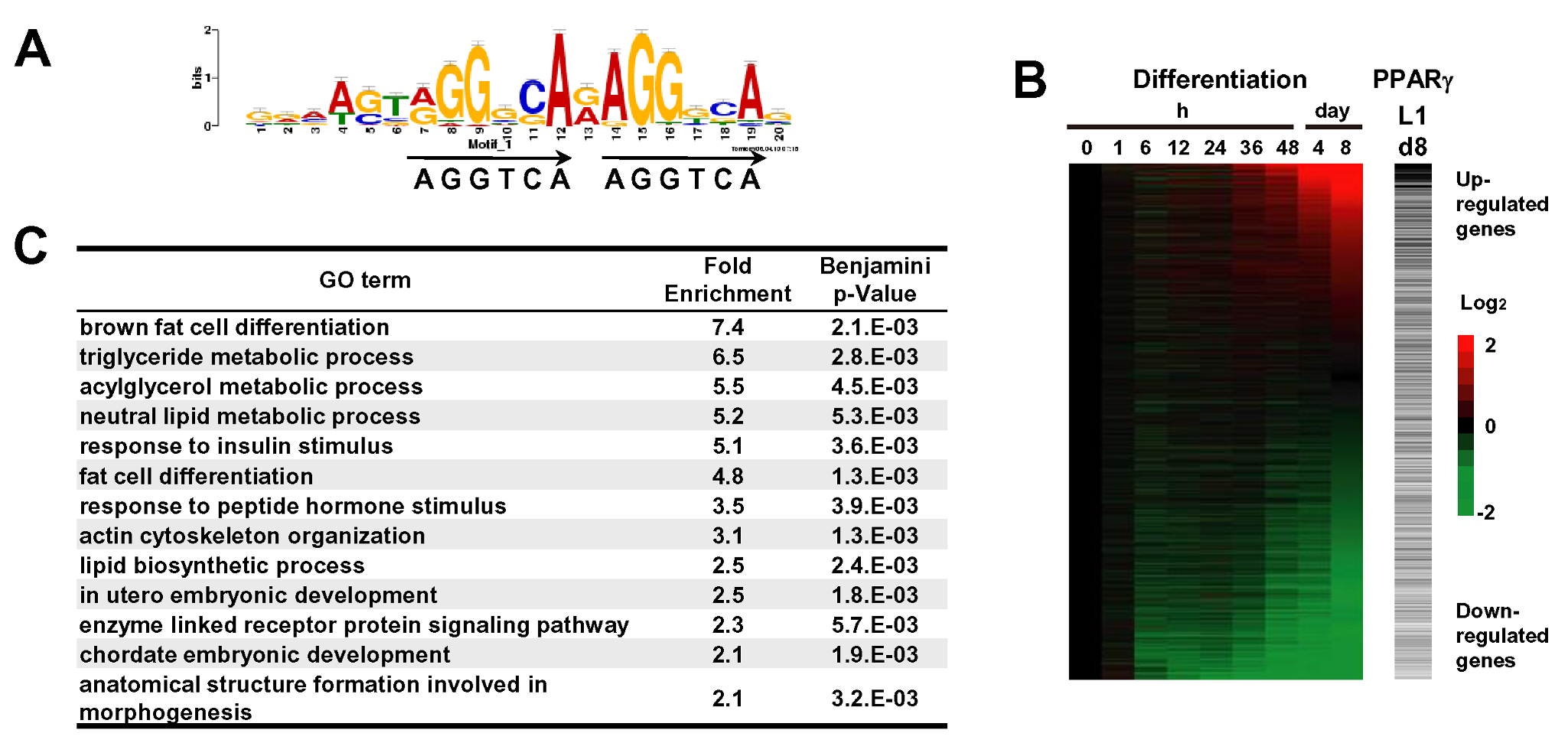

Supplement: Figure S4 — Binding sites for PPARγ and RXRα in 3T3-L1 cells. (A) De novo motif analysis (MEME) of the center 150 bp of the PPARγ/RXRα binding regions (top 400) in 3T3-L1, day 8. Of note, there is a 5′ extension AGT, which corresponds to the interaction between the PPARγ hinge region and DNA identified by crystal structure analysis [41]. (B) A heat map showing enrichment of PPARγ in the vicinity of genes up-regulated during differentiation. The horizontal bars in the right panel indicate each gene bound by PPARγ (+/−25 kb from TSS, day 8) (C) Ontology analysis with DAVID of genes bound by PPARγ [13]. (TIF) [file pgen.1002311.s004.tif]

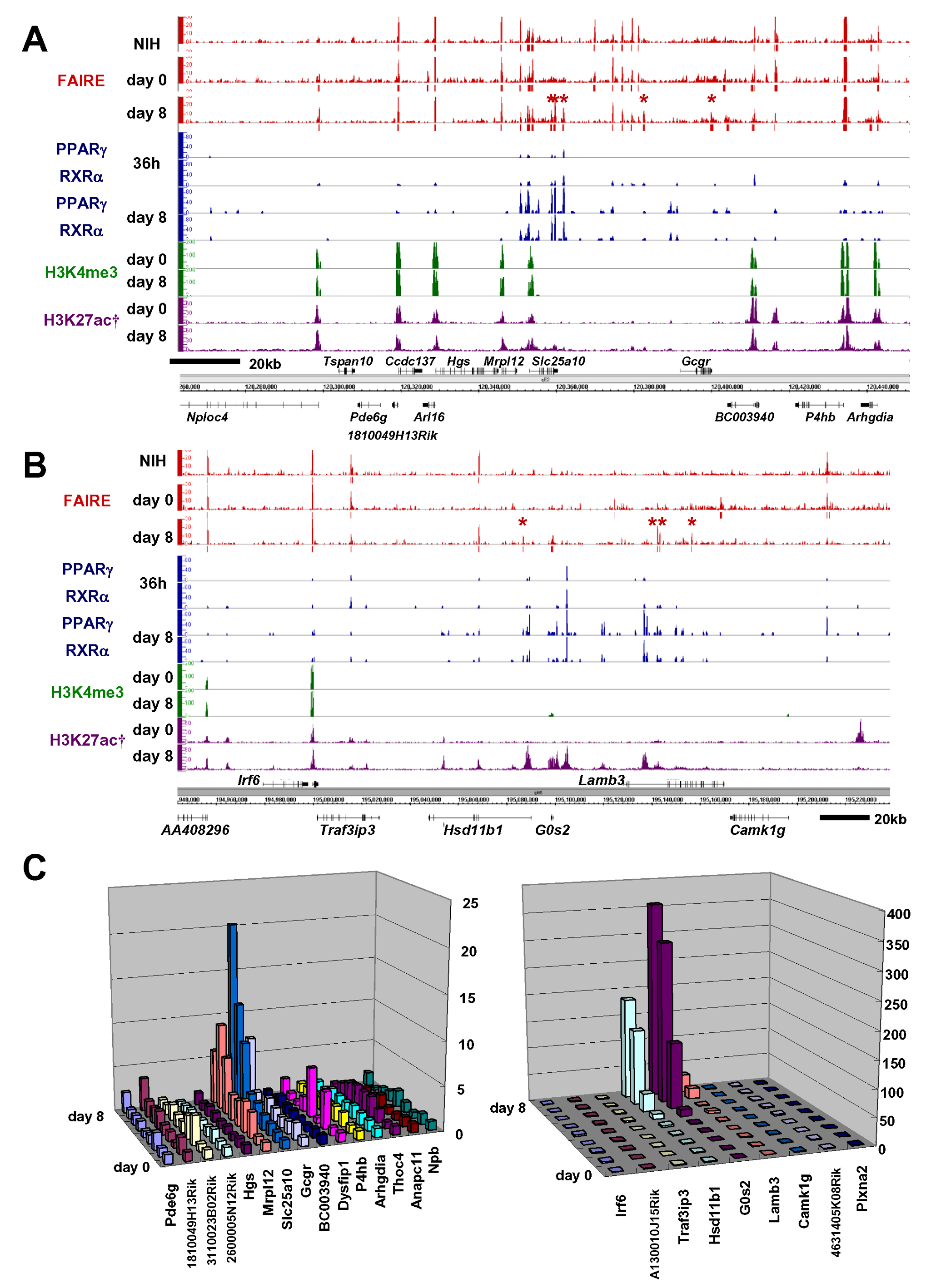

Supplement: Figure S5 — Co-regulation of neighboring genes during adipocyte differentiation. (A, B) Genomic loci near (A) co-regulated Mrpl12, Slc25a10 and Gcgr and (B) co-regulated Hsd11b1, G0s2 and Lamb3. Note, there are clusters of the adipocyte-specific FAIRE peaks (asterisks) and the PPARγ binding sites encompassing the co-regulated genes. (C) Microarray analysis showing co-regulation of Mrpl12, Slc25a10 and Gcgr, and co-regulation of Hsd11b1, G0s2 and Lamb3 included in the clusters of multiple adipocyte-specific FAIRE peak and PPARγ binding sites. (TIF) [file pgen.1002311.s005.tif]

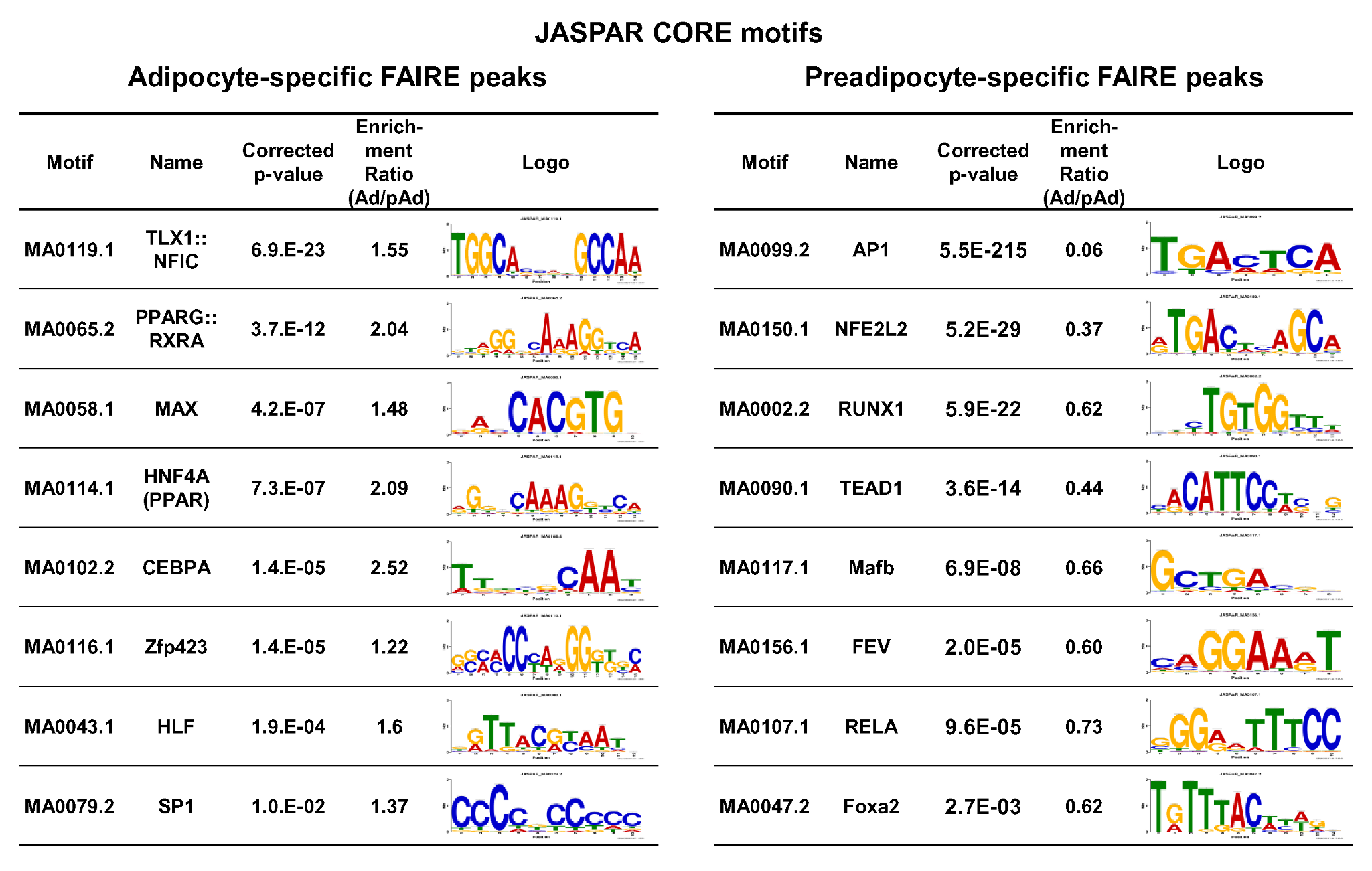

Supplement: Figure S6 — Known motif enrichment analysis of the adipocyte- or preadipocyte-specific FAIRE peaks (JASPAR CORE motifs). Enrichment analysis of the adipocyte- (left) and the preadipocyte-specific (right) FAIRE peaks for known motifs in the JASPAR CORE database performed with AME in the MEME suite by the same methods used in Figure 5. (TIF) [file pgen.1002311.s006.tif]

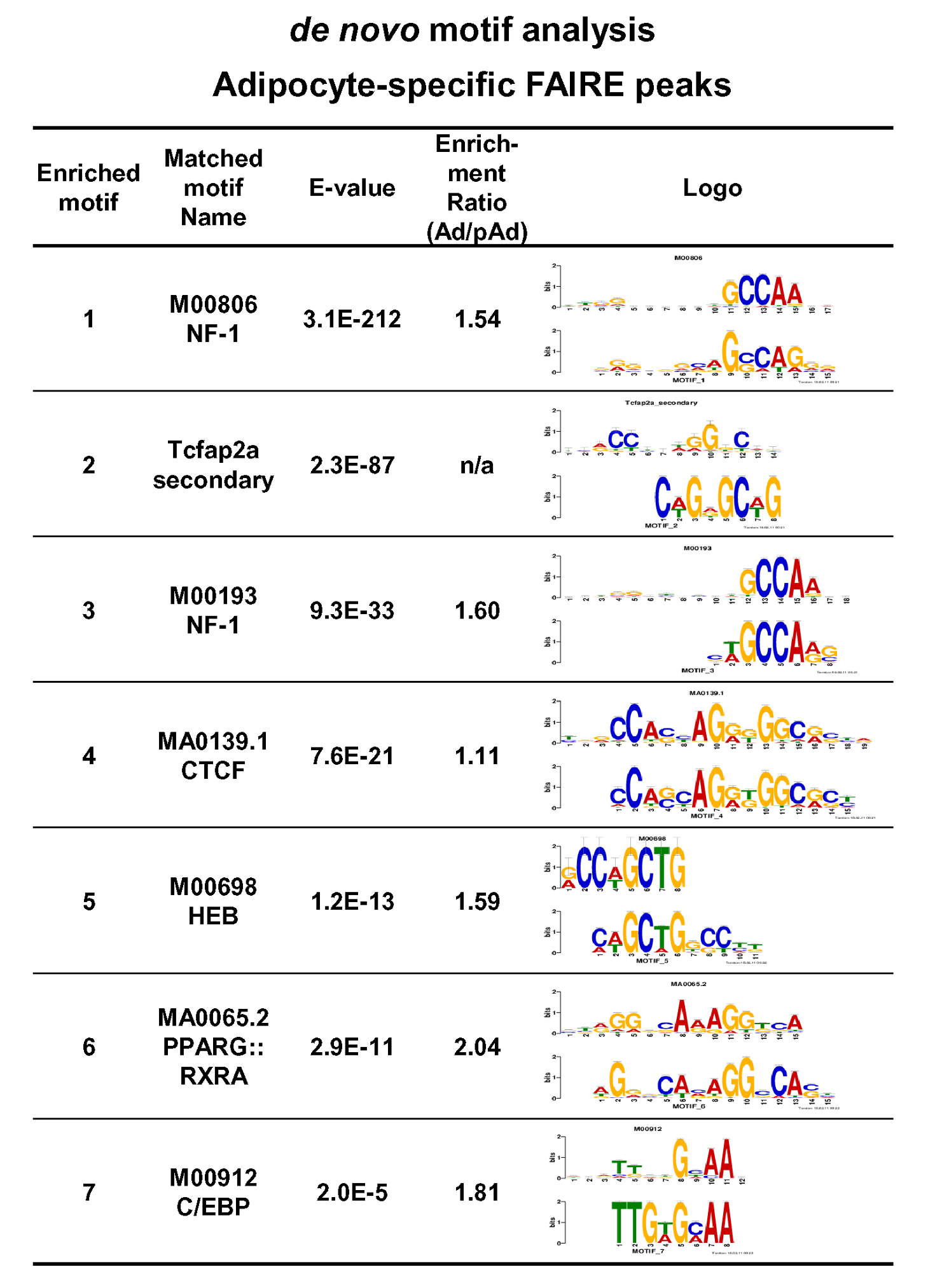

Supplement: Figure S7 — De novo motif analysis of the adipocyte-specific FAIRE peaks. MEME ver. 4.3.0 was used to identify de novo motifs over-represented in the adipocyte- and preadipocyte-specific FAIRE peaks and PPARγ binding sites. After removing repeat regions, DNA sequences from the center 150 bp regions of top 800 cell type–specific FAIRE peaks with higher signals were used for the analyses. Identified enriched de novo motifs were analyzed by TOMTOM in the MEME suite for comparison against a database of known motifs. (TIF) [file pgen.1002311.s007.tif]

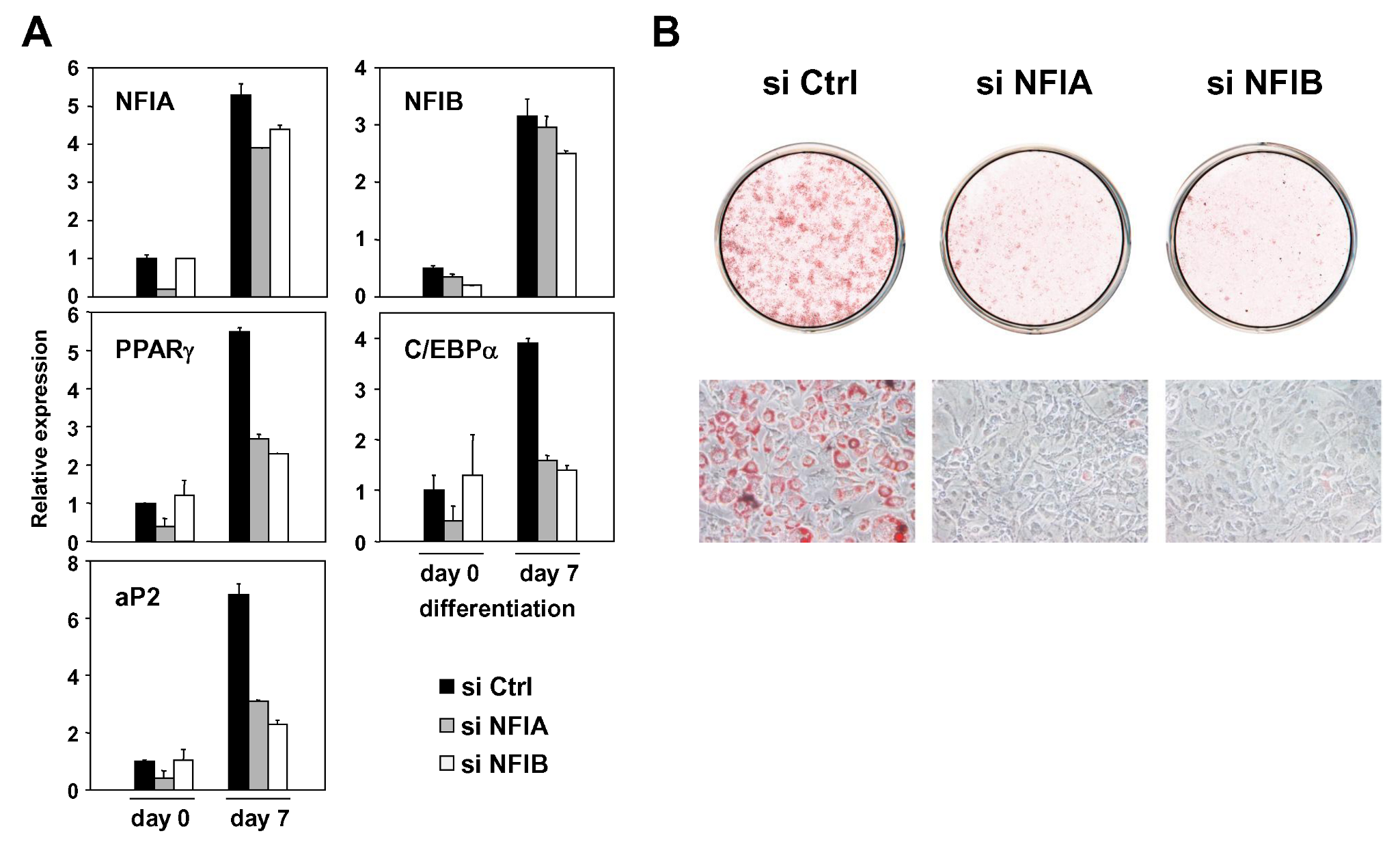

Supplement: Figure S8 — Suppression of adipocyte differentiation by knockdown of NFIA and NFIB by using different siRNAs. (TIF) [file pgen.1002311.s008.tif]

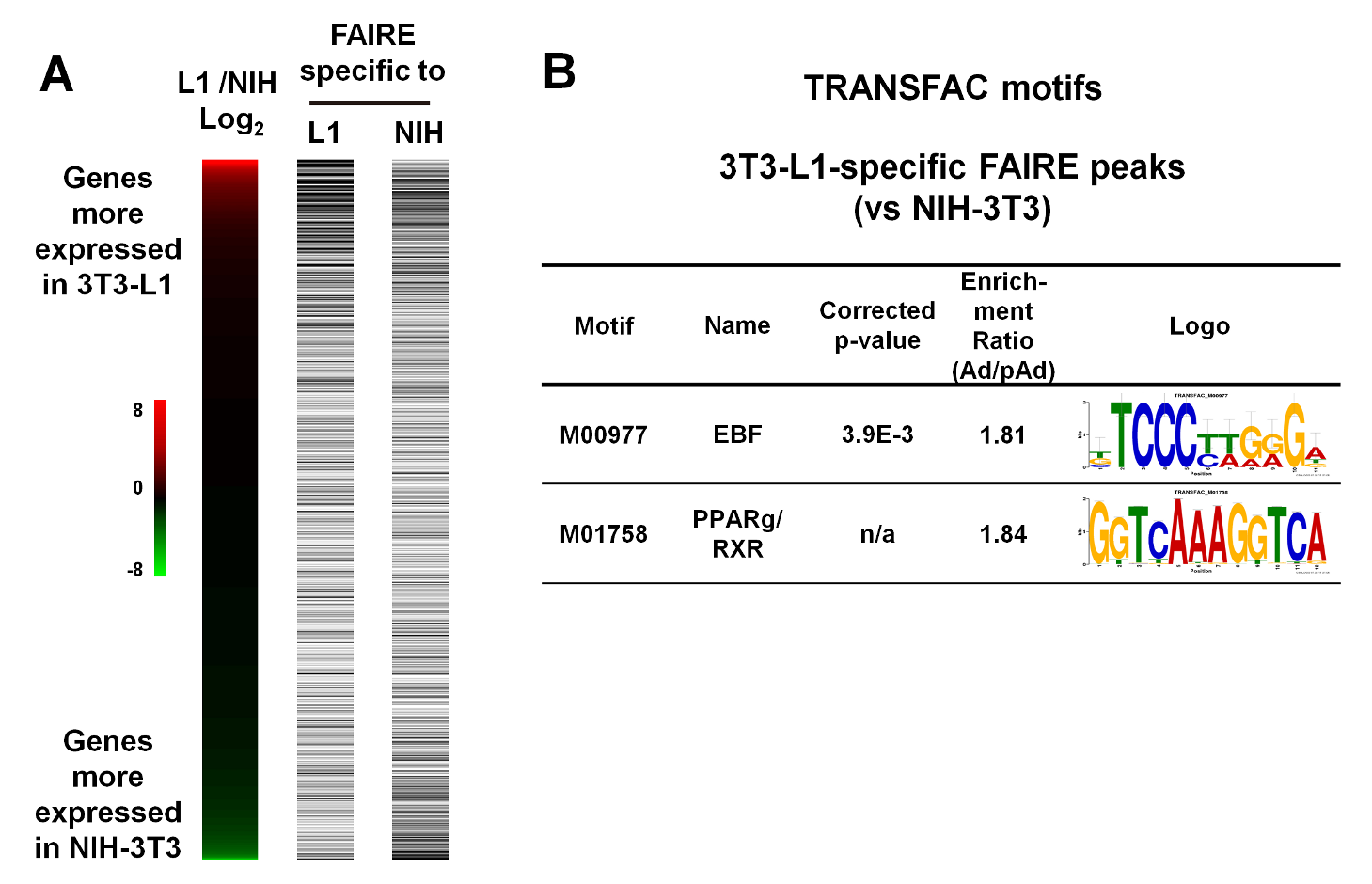

Supplement: Figure S9 — Comparison of FAIRE Peaks between undifferentiated 3T3-L1 and NIH-3T3 cells. (A) A heat map showing enrichment of the 3T3-L1- and NIH-3T3-specific FAIRE peaks in the vicinity (+/−25 kb from TSS) of genes sorted by using the ratio of expression levels in 3T3-L1 or NIH-3T3. The FAIRE peaks specific to 3T3-L1 or NIH-3T3 were enriched in the vicinity of genes whose expression levels were higher in 3T3-L1 or NIH-3T3, respectively. (B) Known motif analysis of the 3T3-L1-specific FAIRE peaks (vs NIH-3T3). The binding motif for EBF and PPARγ/RXR were among the top scored motifs. (TIF) [file pgen.1002311.s009.tif]

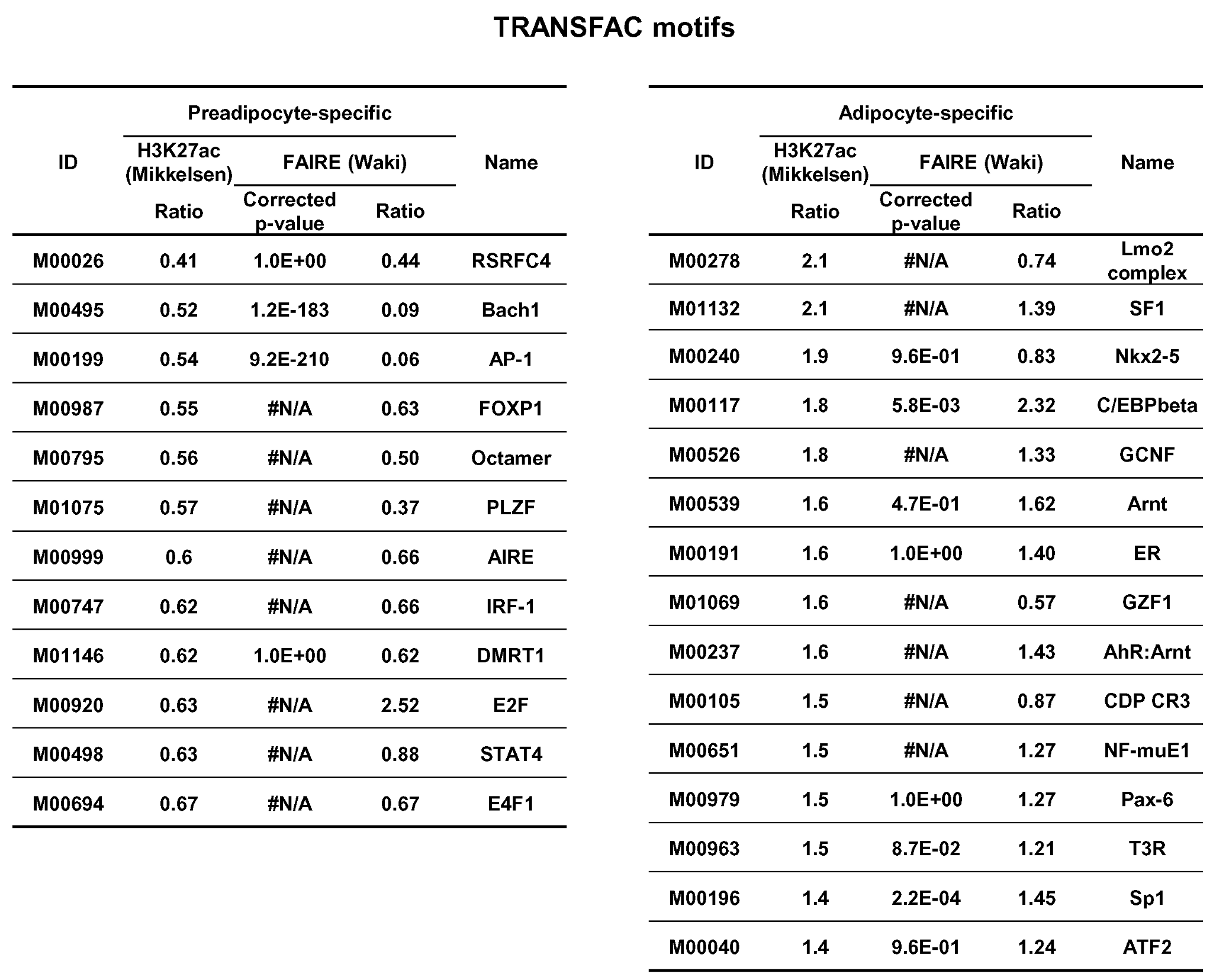

Supplement: Figure S10 — The enrichment ratios of the top motifs in Mikkelsen's study [28] by using the adipocyte- and preadipocyte-specific FAIRE peaks. (TIF) [file pgen.1002311.s010.tif]

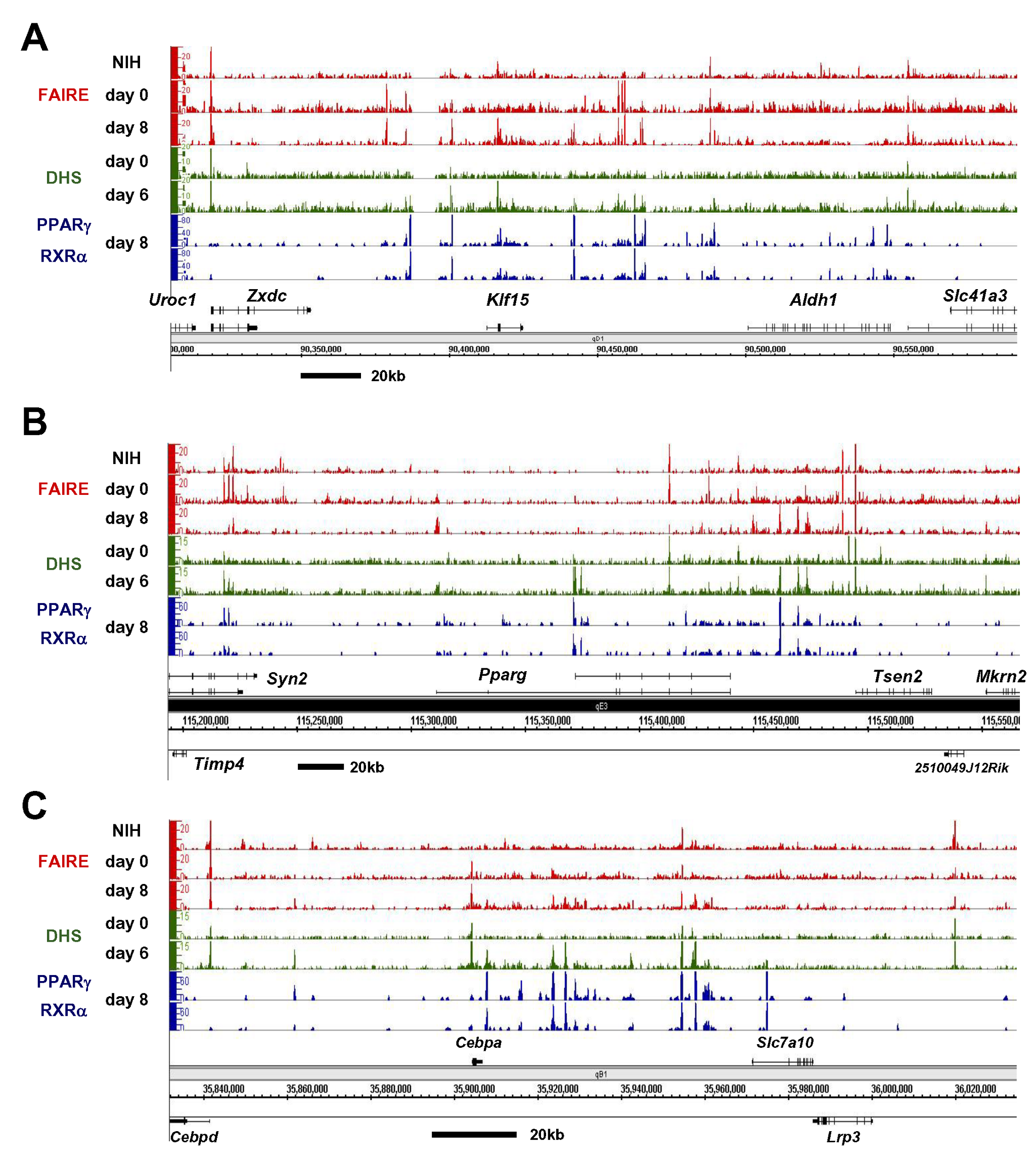

Supplement: Figure S11 — Comparison of DNase-seq in Siersbæk's study [6] and FAIRE-seq peaks near Klf5, Pparg and Cebpa gene. DHS stands for DNase I hypersensitive sites. (TIF) [file pgen.1002311.s011.tif]
