# Supplementary material for: Global Mapping of Cell Type–Specific Open Chromatin by FAIRE-seq Reveals the Regulatory Role of the NFI Family in Adipocyte Differentiation
Source: PLoS Genet. 2011 Oct 20;7(10):e1002311. doi: 10.1371/journal.pgen.1002311 (PMC3197683; doi:10.1371/journal.pgen.1002311)
Supplement: Table S1 — Sequences of primers. (DOC) [file pgen.1002311.s012.doc]

**Table S1. Sequences of Primers**

| qPCR | murine | NFIA_Fwd | ccattttacacaggccaagg |
| --- | --- | --- | --- |
| qPCR | murine | NFIA_Rev | tggctgggtgtgagaagtaag |
| qPCR | murine | NFIB_Fwd | ccggaatacctggagtcg |
| qPCR | murine | NFIB_Rev | gaaatggcaacggtgagg |
| qPCR | murine | NFIA including DN Fwd | ccggaatacctggagtcg |
| qPCR | murine | NFIA including DN Rev | gaaatggcaacggtgagg |
| qPCR | murine | NFIC_Fwd | gatgtattcctccccgctct |
| qPCR | murine | NFIC_Rev | aaccaggtgtaggcgaagg |
| qPCR | murine | NFIX_Fwd | ttctgaacatcccacagcag |
| qPCR | murine | NFIX_Rev | cccattttgaggaacctcttt |
| qPCR | murine | 36B4_Fwd | gatgcccagggaagacag |
| qPCR | murine | 36B4_Rev | acaatgaagcattttggataatca |
| qPCR | murine | aP2_Fwd | caccgcagacgacaggaag |
| qPCR | murine | aP2_Rev | gcacctgcaccagggc |
| qPCR | murine | PPARγ_Fwd | ccattctggcccaccaac |
| qPCR | murine | PPARγ_Rev | Aatgcgagtggtcttccatca |
| qPCR | murine | C/EBPα_Fwd | ccttcaacgacgagttcctg |
| qPCR | murine | C/EBPα_Rev | tggccttctcctgctgtc |
| qPCR | murine | C/EBPβ_Fwd | aagagccgcgacaaggc |
| qPCR | murine | C/EBPβ_Rev | gtcagctccagcaccttgtg |
| qPCR | murine | C/EBPδ_Fwd | tgcccaccctagagctgtg |
| qPCR | murine | C/EBPδ_Rev | cgctttgtggttgctgttga |
| Cloning | murine | EcoRI NFIA Fwd | aaaaaagaattcgccaccatgtattctccgctctgtct |
| Cloning | murine | XhoI NFIA Rev | aaaaaactcgagttatcccaggtaccaggact |
| Cloning | murine | BamHI NFIB Fwd | aaaaaaggatccgccaccatgatgtattctcccatctgtctc |
| Cloning | murine | NotI NFIB Rev | aaaaaagcggccgctcagttgcttgtctccgcttgaag |
| Cloning | murine | XhoI NFIA DN Rev | aaaaaactcgagttaagcttcacttggctggctgg |
| ChIP | murine | B1 Fwd | aagtcctttgtttccctccttc |
| ChIP | murine | B1 Rev | ggcctatacttaggcagcatgt |
| ChIP | murine | B2 Fwd | ggagttccctctcattccttct |
| ChIP | murine | B2 Rev | caggacttcaggtggaattgtt |
| ChIP | murine | Site_1 Fwd | catcagagaggcagaaaaacg |
| ChIP | murine | Site_1 Rev | cttggagccatatccctcttc |
| ChIP | murine | Site_2 Fwd | gccaagagtcgcagtctcag |
| ChIP | murine | Site_2 Rev | gtatgtggccaagagtctgga |
| ChIP | murine | Site_3 Fwd | ctgacagcccctttcctaact |
| ChIP | murine | Site_3 Rev | ggaggctgagtcttggctaat |
| ChIP | murine | Site_4 Fwd | gctcaccaggtgtgaggaa |
| ChIP | murine | Site_4 Rev | aatcccaagccataaagcac |
| ChIP | murine | Site_5 Fwd | actgcatccagcaaggagat |
| ChIP | murine | Site_5 Rev | ctccggggcttttctttact |
| ChIP | murine | Site_6 Fwd | cttagagaagggcgttgtgc |
| ChIP | murine | Site_6 Rev | cagcttttccaaggcatgtt |
| ChIP | murine | Site_7 Fwd | gacacaaggcacatggacac |
| ChIP | murine | Site_7 Rev | acccttgacccaaactgttg |
| ChIP | murine | Site_8 Fwd | tggggctctgttttatttgc |
| ChIP | murine | Site_8 Rev | gccgtgtttctctcctgaac |
| ChIP | murine | Site_9 Fwd | tgggaactccatttgctctc |
| ChIP | murine | Site_9 Rev | gaacagaattcccagcagga |
| ChIP | murine | Site_10 Fwd | tagatgacaaccgccacaac |
| ChIP | murine | Site_10 Rev | gttactgcctgacggttggt |
| ChIP | murine | Site_11 Fwd | ggcaagtagccaaatgaacc |
| ChIP | murine | Site_11 Rev | tctgtcctagaagttttctcaaagc |
